# Divergent endophytic viromes and phage genome repertoires among banana (*Musa*) species

**DOI:** 10.3389/fmicb.2023.1127606

**Published:** 2023-06-09

**Authors:** Shiva A. Aghdam, Rachel M. Lahowetz, Amanda M. V. Brown

**Affiliations:** Department of Biological Sciences, Texas Tech University, Lubbock, TX, United States

**Keywords:** viral community, bacteriophage, endogenous virus, *Musa*, diversity, microbiome

## Abstract

**Introduction:**

Viruses generally cause disease, but some viruses may be beneficial as resident regulators of their hosts or host microbiomes. Plant-associated viruses can help plants survive by increasing stress tolerance or regulating endophytic communities. The goal of this study was to characterize endophytic virus communities in banana and plantain (*Musa* spp.) genotypes, including cultivated and wild species, to assess virome repertoires and detect novel viruses.

**Methods:**

DNA viral communities were characterized by shotgun sequencing of an enriched endosphere extract from leaves and roots or corm of 7 distinct *Musa* genotypes (*M. balbisiana*, Thai Black, *M. textilis*, *M. sikkimensis*, Dwarf Cavendish, Williams Hybrid, and FHIA-25 Hybrid).

**Results:**

Results showed abundant virus-like contigs up to 108,191 bp long with higher relative abundance in leaves than roots. Analyses predicted 733 phage species in 51 families, with little overlap in phage communities among plants. Phage diversity was higher in roots and in diploid wild hosts. *Ackermanniviridae* and *Rhizobium phage* were generally the most abundant taxa. A *Rhizobium* RR1-like phage related to a phage of an endophytic tumor-causing rhizobium was found, bearing a holin gene and a partial Shiga-like toxin gene, raising interest in its potential to regulate endophytic Rhizobiaceae. *Klebsiella* phages were of interest for possible protection against Fusarium wilt, and other phages were predicted with potential to regulate *Erwinia*, *Pectobacterium*, and *Ralstonia*-associated diseases. Although abundant phage-containing contigs were functionally annotated, revealing 1,038 predicted viral protein domains, gene repertoires showed high divergence from database sequences, suggesting novel phages in these banana cultivars. Plant DNA viruses included 56 species of *Badnavirus* and 26 additional non-*Musa* plant viruses with distributions that suggested a mixture of resident and transient plant DNA viruses in these samples.

**Discussion:**

Together, the disparate viral communities in these plants from a shared environment suggest hosts drive the composition of these virus communities. This study forms a first step in understanding the endophytic virome in this globally important food crop, which is currently threatened by fungal, bacterial, and viral diseases.

## Introduction

Pathogenic plant viruses have received much attention; however, some viruses can play important beneficial roles. For example, various DNA and RNA viruses appear to help plants survive restrictive environmental conditions and increase biotic and abiotic stress tolerance (Schoelz and Stewart, 2018; Seo and Kweon, 2019; Pratama et al., 2020; Siddique, 2020). Moreover, interactions between viruses, microbes, and hosts can create complex dynamics (Knief et al., 2012; Van Belleghem et al., 2018; Rodriguez et al., 2019) in which viruses may serve as resident regulators impacting hosts or their endophytic populations to alter net secondary metabolite biosynthesis (Roossinck et al., 2015; Seo and Kweon, 2019). Using whole-genome shotgun genomics (WGS) can reveal hidden viral diversity within plants (Forero-Junco et al., 2021). However, most endophyte WGS studies focus on bacterial or fungal communities, with limited analysis of DNA viruses (Regalado et al., 2020; Akinola et al., 2021; Soto-Giron et al., 2021; Fadiji et al., 2021a,b).

Among plant virome communities, bacteriophage (hereafter phage) communities may be the least studied but most impactful on host biology (Dion et al., 2020). Phages may impact hosts by controlling bacterial diseases or modifying beneficial bacterial communities (Buttimer et al., 2017; Koskella and Taylor, 2018; Morella et al., 2018; Koskella, 2019). Using phages as a therapy against pathogenic bacteria has been widely explored (Tian et al., 2015; Addy et al., 2016; Murthi et al., 2021), with some studies indicating plant bacterial diseases may be efficiently controlled by phages (Koskella and Taylor, 2018). For example, onion leaf blight caused by *Xanthomonas axonopodis* pv. *allii* showed a 50% reduction in disease severity when phages were applied weekly or biweekly (Lang et al., 2007). Peach bacterial spot caused by *X. campestris* pv. *pruni* was controlled by spraying trees with phages (Saccardi et al., 1993). Impacts of phages on beneficial bacteria in animal guts (Federici et al., 2021) and in soils (Kuzyakov and Mason-Jones, 2018; Blazanin and Turner, 2021) are well-established, but few studies have examined phage effects within plant microbiomes, although at least one study has shown that phages can alter phyllosphere diversity (Morella et al., 2018).

Plant dsDNA viruses, many of which alternate between circulating infectious virions and integrated endogenous virus elements (EVEs), are also inadequately studied (Gayral et al., 2008; Flynn and Moreau, 2019; Tripathi et al., 2019). Some EVEs can be activated and lead to systemic infection of the host when the plants are stressed (Muller et al., 2021), while others are ‘molecular fossils’ in domesticated plants (Jones et al., 1999). Latent plant viruses can benefit host plants (Pagán et al., 2012; Tripathi et al., 2019) by contributing to plant virus resistance through induction of transcriptional or post-transcriptional gene silencing of homologous sequences (Harper et al., 2002). The genomes of bitter orange (*Poncirus trifoliata*), potato (*Solanum tuberosum*), rice (*Oryza sativa*), tomato (*Lycopersicon* sp.), petunia (*Petunia* sp.), tobacco (*Nicotiana* sp.), and banana (*Musa* spp.) have been shown to harbor such integrates. Banana streak Obino l’Ewai virus has severely hindered international banana (*Musa* spp.) breeding programs, as new hybrids become infected through the integrated viruses from the wild *M. balbisiana* (BB genotype; Geering et al., 2005). Insertion of a badnavirus promoter next to an endogenous plant gene may change its transcription levels and alter the tissue specificity of expression, hindering banana breeding programs (Matzke et al., 2004).

Banana and plantain (*Musa* spp.) are a model group for which natural endophytic viromes have not been well-characterized. Banana is an important food staple and commercial product in over 130 countries (FAO, 2016). While common pathogenic viruses, such as banana streak virus (BSV), banana bunchy top virus (BBTV), and banana bract mosaic virus (BBrMV) have been studied extensively (Dale, 1987; Bateson and Dale, 1995; Lockhart, 1995), natural viral community variance is not well understood. Although plant tissue and cultivar shape microbiomes (Hirsch and Mauchline, 2012), the host impact on plant viromes is less clear (Koskella and Taylor, 2018; Harrison and Griffin, 2020). Furthermore, domestication appears to deplete bacterial and fungal microbiomes (Xue et al., 2015; Köberl et al., 2017), but its impacts on banana viromes are unknown.

The current study examined DNA viruses co-collected with enriched microbiomes from *Musa* spp. grown in sympatry, to assess the impacts of hosts on viral communities. Comparing viromes from endophytic tissues of both roots and leaves of wild *Musa* diploid genotypes (*Musa balbisiana, Musa textilis*, and *Musa sikkimensis*) and domesticated triploid genotypes (Dwarf Cavendish, Williams Hybrid, and FHIA-25 Hybrid), we identified putative phages and endogenous viral elements, illuminating distinct community profiles and predicted numerous potentially novel DNA viruses.

## Materials and methods

### Sample collection

To compare endophytic virus-like sequences to estimate viral communities from different banana (*Musa* spp.) genotypes, seven cultivars and species of banana were collected from a farm in Homestead, Florida (Table 1). While some plants at the farm appeared to have low-level symptoms suggesting the presence of Black sigatoka (*Mycosphaerella fijiensis*) disease, the farm used standard intermittent control measures to limit this and other pathogens. Collected plants used in this study appeared to be symptom-free before, during, and after collection. To assess the differences in viruses present in above-ground and below-ground tissues, two samples were taken from each plant: one from the leaves and another from the root or corm of the same plant. Plant samples were first washed in tap water for 10 min to remove any loose material. Then tissues were surface-sterilized (Refaei et al., 2011) by immersing them for 1 min in 70% ethanol, then for 3 min in 2.5% sodium hypochlorite solution, followed by 1 min in 70% ethanol and finally rinsed three times with sterile distilled water.

**Table 1:** Banana (*Musa* spp.) sampled in this study, showing species or cultivar name, genotype, tissues sampled, and domestication status.

Sample name	Tissue	Species	Cultivar	A/B genotype	Type
WHL	Leaf	*Musa acuminata*	Williams Hybrid	AAA	Domesticated
WHC	Corm				
DCL	Leaf	*Musa acuminata*	Dwarf Cavendish	AAA	Domesticated
DCR	Root				
MBL	Leaf	*Musa balbisiana*	*Musa balbisiana*	BB	Wild
MBR	Root				
BBL	Leaf	*Musa balbisiana*	Thai Black	BB	Wild
BBC	Corm				
FHL	Leaf	*Musa acuminata*	FHIA-25 Hybrid	AAB	Domesticated
FHC	Corm				
MTL	Leaf	*Musa textilis*	*Musa textilis*	N/A	Wild
MTC	Corm				
MSL	Leaf	*Musa sikkimensis*	*Musa sikkimensis*	N/A	Wild
MSR	Root				

### Culture-free enrichment for microbial endophytes and their viromes

To reduce sequence yield of non-target plant genomes while obtaining microbial endophytes and potentially their associated viruses, we used a culture-free filtration and density gradient method previously used for soybean (Ikeda et al., 2009). One of the goals here was to examine for the first time whether this method could recover plant and endophyte DNA viromes while recovering cellular endophytic microbiota. Briefly, the method used 100 g of each plant sample, which was homogenized in a sterilized blender in 400 mL of ice-cold “BCE” buffer (50 mM Tris–HCl pH 7.5, 1% Triton X-100, 2 mM 2-mercaptoethanol). To preserve cell and DNA integrity, all steps were performed at 4°C. Resulting homogenate was filtered through a UV-sterilized Miracloth (EMD Millipore) rayon-polyester mesh, then centrifuged for 5 min at 500xg. Flow-through was transferred to sterile tubes and centrifuged for 20 min at 5,000× g. Resulting supernatant was discarded and pellets were resuspended in 50 mL of BCE. This slurry was then filtered through a UV-sterilized Kimwipe (Kimberly-Clark) and the filtrate was centrifuged for 10 min at 5,000× g. Resulting supernatants were discarded and pellets were resuspended in 35 mL of BCE. This Kimwipe filtration was repeated. Final pellets were resuspended in 6 mL of 50 mM Tris–HCl (pH 7.5). Each 1 mL aliquot was pipetted over 4 mL of Nycodenz® (3.2 g of Nycodenz® + 4 mL of 50 mM Tris–HCl, pH 7.5), then gradients were centrifuged for 40 min at 5,000× g to collect a layer of microbial cells at the interface. While this method may result in pelleting of free viral particles; our goal was to see whether virome members such as phages associated with bacteria or viruses associated with plants/fungi may be retained in this microbiome layer.

### DNA extraction, Illumina library preparation, and sequencing

DNA was isolated from the enriched microbial layers using the DNeasy Blood and Tissue Kit from QIAGEN (Valencia, CA) following the manufacturer’s directions. DNA quantity and quality were assessed on the Nanodrop spectrophotometer. Shotgun metagenomic libraries were prepared from ~0.5 to 1 μg of DNA with the QIAseq FX 96 DNA Library Kit (Valencia, CA). Libraries were checked for quality on the TapeStation 2200 (Agilent) then normalized and pooled for sequencing. Sequencing was performed at the Center for Biotechnology and Genomics (CGB) at Texas Tech University and Genewiz, Inc. (NJ) with Illumina paired-end reads of 105 and 150 bp, respectively.

### Quality filtering, assembly, and initial blast to virus databases

Paired-end read overlaps were merged using PEAR v0.9.1 (Zhang et al., 2014), then Trimmomatic v0.38 (Bolger et al., 2014) was used to trim and filter reads prior to assembly with metaSPAdes in the SPAdes software v3.13.0 (Bankevich et al., 2012; Nurk et al., 2017), with read-error correction and using kmers 21, 33, 45, 59, 73, and 99. Assembly statistics were calculated using QUAST v5.0. (Gurevich et al., 2013). Initial classification of taxa was performed by using DIAMOND v2.0.9 (Buchfink et al., 2014) blastx on assembled contigs against the NCBI nr database (accessed December 2020) with options sensitive, minimum e-value 1e-05, block size 16 and single chunk used for the seed index. Reads were mapped to the taxon-annotated contigs using BWA-MEM to assess relative abundances and normalized for relative average genome sizes among groups (e.g., banana haploid genome 523 Mbp, fungi 40 Mbp, bacteria/archaea 4 Mbp, viruses 60 kbp). All contigs were then searched for virus-like regions using a more sensitive custom two-part blastn approach, as follows. First, to generate a custom viral nucleotide blast database consisting of 3,250,606 viral sequences, all viral sequences on NCBI were downloaded (db date 02 January 2020) to generate a custom viral nucleotide blast database consisting of 3,250,606 viral sequences. Then, assembled contigs were analyzed against this custom virus database with blastn in BLAST+ v2.10.0 (Camacho et al., 2009) with the following parameters: e-value cutoff 10, maximum target sequences 500, keeping all blastn hits for the next step. Fasta sequences for all initial blastn hits were trimmed to only the hit regions using samtools faidx (Li et al., 2009), and resulting virus-like regions were subjected to a second blast to the full NCBI nucleotide database (nt) with e-value cutoff 0.01 and maximum target sequences 20, saving only contigs with top bitscore matching virus, phage, or prophage/provirus in the taxonomic field or elsewhere in the text of the blast hit.

### Refined virus identification, classification, trimming of host regions, and deduplication

To more sensitively and rigorously search for viruses, we employed recently validated virus identification pipelines (Gregory et al., 2019; Pratama et al., 2021), using assembled contigs as input and performing an initial virus scan using VirSorter2 v.2.2.1 (Roux et al., 2015; Guo et al., 2021), then assessing quality and completeness of the VirSorter2 outputs using CheckV (Nayfach et al., 2021), trimming any host DNA before repeating VirSorter2 with modified parameters. For the initial VirSorter2, we used a loose cutoff of 0.5 for maximal sensitivity with minimal length 500 bp, and limited the search to dsDNA and ssDNA phage. CheckV analysis of VirSorter2 outputs used minimal score cutoff of 0.5. Resulting viral contigs were run in VirSorter2 with a minimum score of 0.9 to detect more confident contigs, using parameters --seqname-suffix-off and --viral-gene-enrich-off. Next, potential duplications in the form of populations of similar viral fragments were removed using the deduplication program dRep (Olm et al., 2017) with parameters -con 10 and -sa 0.95. Dereplicated contigs were then taxonomically classified by two methods shown recently to give slightly different results (Lee et al., 2022): vConTACT2 (Bin Jang et al., 2019) which uses a distance-based hierarchical clustering; and Genome Detective Virus Tool (GDV) v.2.52 (Vilsker et al., 2019). GDV virus annotations were classified to higher taxonomic levels by referencing NCBI taxonomy databases (“fullnamelineage.dmp” downloaded April 2023).

### Viral abundance, community overlap, and diversity analysis

The relative abundance of each predicted virus in each sample was estimated based on kmer coverage of each contig matched to a virus in blast or with vConTACT2 or Genome Detective Virus Tool (Supplementary Table 1), modified by the following equation: C = (C_K_ R)/(R − K + 1), where C is absolute coverage, C_K_ is kmer coverage estimated by metaSPAdes, K is the length of kmers used in metaSPAdes, and R is read length. Before analysis of diversity, alpha rarefaction curves were generated from total reads not mapped to *Musa* spp. (i.e., from reads derived from the microbiome fraction). For samples with sufficient data based on asymptote in rarefaction curves, data were then normalized to adjust for differences in sequencing effort among samples as follows: (a) for phages, this normalization was performed by scaling each sample’s coverage per contig to the highest sample read count from reads that were not mapped to *Musa* spp., under the premise that phage abundance should be linked to microbiome abundance rather than plant DNA (see right column in Supplementary Table 2), or (b) for endogenous viruses, this normalization was scaled to the coverage to the highest sample read count from reads that mapped to *Musa* spp. (see second to right column in Supplementary Table 2), under the premise that endogenous virus abundance should be linked to host plant DNA levels. Relative abundances were plotted as heatmaps. Viral community overlap among samples was depicted with the proportional Venn drawing tool nVenn (Pérez-Silva et al., 2018). Alpha diversity analysis was performed in R to assess Shannon diversity with the Hutcheson *t*-test using “ecolTest” v. 0.0.1 (Hutcheson, 1970; Zar, 2010).

### Annotation and analysis of complete or fragmented phages and prophages

Basic initial gene annotation of virus-like contigs identified either by the two-part blast beginning with the virus database or by the DIAMOND blast described above were annotating in Prokka v.1.14.6 (Seemann, 2014) using the parameter—kingdom Viruses. Prokka uses Prodigal for *ab initio* gene prediction, HMMER3 for protein family profiles, and BLAST+ for comparative annotation. A more refined virus-specific gene annotation was performed on the output viral contigs from the VirSorter2-dRep-GDV pipeline. This functional annotation used the DRAM-v software (Shaffer et al., 2020) using the parameter -min_contig_size 1,000.

A small subset of longer contigs were examined for orthologs and gene synteny as follows. Annotated viral gene orthologs were determined using Roary v3.13.0 (Page et al., 2015) on gff outputs from Prokka, with parameters -e for codon-aware alignment in PRANK (Löytynoja, 2014) and -i 50 to detect distant orthologs. Annotated contigs were then imported into Geneious Prime v2020.0.4 (Biomatters, Ltd), and subjected to between-sample assembly using the Geneious *de novo* assembler with default parameters. Resulting contigs were analyzed using the online platform PHASTER (Zhou et al., 2011; Arndt et al., 2016). Output phage/prophage were re-analyzed with DIAMOND blastx to predict taxonomy and function of phage-like regions. For the two longest genes in these phages, phylogenetic analysis was performed. First, blastn to the nt database at NCBI was used to find and download homologous genes. These homologs were then aligned in Geneious with CLUSTAL Omega, and phylogenetic analysis was performed using maximum likelihood (ML) phylogeny reconstruction was performed in RAxML v4.0 (Stamatakis, 2014) using the GTR Gamma nucleotide model, with rate heterogeneity alpha estimated, and with rapid bootstrapping and search for the best-scoring ML tree (−f a -x 1) assessing bootstrap support from 1,000 replicates. Annotated phage or prophage regions were then compared for synteny and similarity to the most similar full-length sequences available in GenBank using Easyfig v2.2.2 (Sullivan et al., 2011).

## Results

### Sequence output and metagenomic assembly statistics

After overlap merging, filtering, and trimming, the total enriched microbiome layers yielded a total of ~576 million high-quality reads from 14 samples (Supplementary Table 2), with a range between 13 and 84 million reads per sample. Metagenomic assemblies from these reads produced a total of ~5 million contigs, with between ~4,000 and ~300,000 contigs per sample with assembly N50s of 670 to 9,897 per sample (Supplementary Table 2). Rarefaction curves indicated sufficient sampling of microbiomes for each genotype, except for leaves of Williams Hybrid, which appeared undersampled and did not have detectable viruses.

### Taxonomic composition of the enriched banana microbiome and its DNA viral community

**Figure 1:**
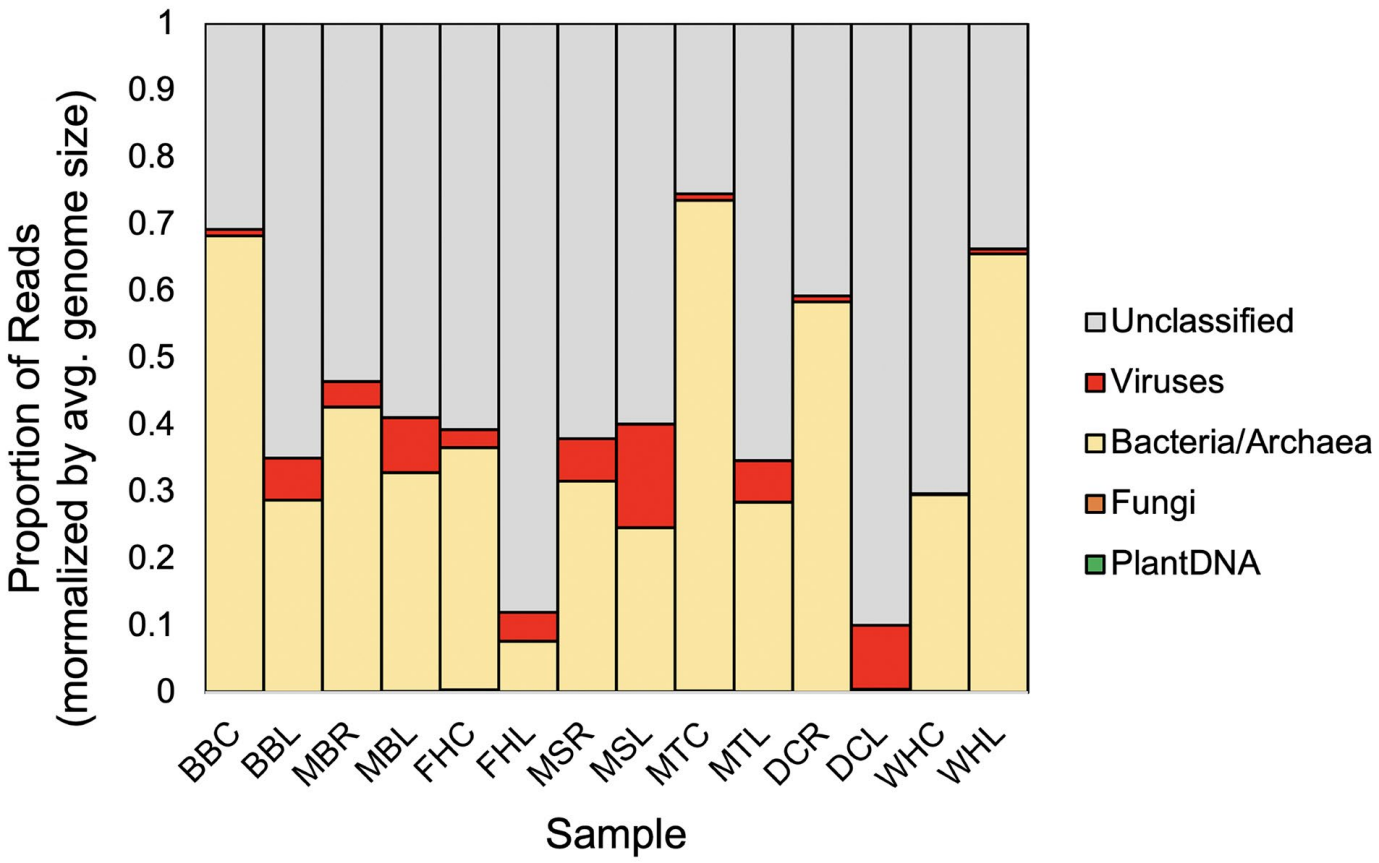
Relative proportion of reads from extracted *Musa* microbiome layers that mapped to plant, fungi, bacteria and archaea, and viruses, controlling for differences in average genome sizes and showing reads mapped to contigs that could not be classified to any taxon. Average genome sizes used: banana haploid genome 523 Mbp, fungi 40 Mbp, bacteria/archaea 4 Mbp, viruses 60 kbp. Samples: BBC and BBL = *Musa balbisiana* Thai Black corm and leaf, DCR and DCL = Dwarf Cavendish root, and leaf, FHC and FHL = FHIA-25 corm and leaf, MBR and MBL = *Musa balbisiana* root and leaf, MSR and MSL = *Musa sikkimensis* root and leaf, MTC and MTL = *Musa textilis* corm and leaf, WHC and WHL = Williams Hybrid corm and leaf.

Initial taxonomic classification of the enriched microbiome layers, using DIAMOND blastx and controlling for average genome sizes showed these microbiomes had very few plant or fungal genomes (0.11 and 0.28%, respectively) compared to bacterial and viral genomes (92.4% and 7.2%, respectively) on average. There were consistently higher relative levels of predicted virus matches in leaves than in roots (Figure 1) and plant genotypes differed slightly in these proportions. Notably, 20.5% of all reads before normalizing for genome sizes could not be assigned to any taxonomic group, even at the kingdom level. It is unknown if these reads contain additional undescribed viruses.

The results of the two-step blastn of assembled contigs to viral databases showed 864 contigs with highest blast hits to viruses, comprising ~1.13 Mbp of assembled sequence length with maximum contig length 53,615 bp (Table 2). Among these, 156 contigs matched phage and 708 contigs matched plant DNA viruses of the family Caulimoviridae, which likely comprise both endogenous (i.e., integrated) sequences such as endogenous pararetroviruses, and episomal (i.e., nonintegrated) viruses. In total, read counts across all samples (non-normalized across samples) at the order level mostly matched Ortervirales (95.16% of reads) which are a broad group containing almost all dsDNA viruses that replicate through an RNA intermediate, followed by Caudovirales (4.8% of reads) which are tailed bacteriophages, and Tubulavirales (0.02% of reads).

**Table 2:** *Musa* samples and their endophytic virus-like sequences matches, showing numbers of contigs resulting from using two different virus detection pipelines (i.e., a blastn-based pipeline and more sensitive pipeline using the software VirSorter2 with vConTACT2 and GDV).

Sample ID	No. of contigs matching putative phage by blastn	No. of contigs matching putative endogenous viruses by blastn	No. of contigs matching putative phage by vConTACT2 + GDV pipeline	Sum of contig lengths matching viruses by vConTACT2 + GDV pipeline
BBC	55	21	68	3,404,449
BBL	9	124	123	1,641,355
DCR	12	32	67	2,173,216
DCL	–	66	–	–
FHC	19	30	81	5,222,084
FHL	2	103	8	948,983
MBR	14	61	77	2,022,359
MBL	6	112	29	2,094,866
MSR	5	31	29	621,307
MSL	3	45	28	174,439
MTC	30	3	156	4,703,559
MTL	2	80	35	831,313
WHC	–	–	32	32,827
WHL	–	–	–	–
Total	156	708	733	23,870,757

Samples: BBC and BBL = *Musa balbisiana* Thai Black corm and leaf, DCR and DCL = Dwarf Cavendish root, and leaf, FHC and FHL = FHIA-25 corm and leaf, MBR and MBL = *Musa balbisiana* root and leaf, MSR and MSL = *Musa sikkimensis* root and leaf, MTC and MTL = *Musa textilis* corm and leaf, WHC and WHL = Williams Hybrid corm and leaf.

By comparison, the more sensitive viral detection pipeline using VirSorter2, vConTACT2, and GDV viral prediction pipelines yielded significantly more candidate viruses (Table 2 and compare Supplementary Tables 1–3), with ~23.870 Mbp of contigs containing predicted viruses, comprising 733 predicted phages. vConTACT2 clustering was able to taxonomically assign 22.51% of the contigs, whereas GDV was able to taxonomically assign 94.27% of viral contigs. Compared to the blastn pipeline, this more sensitive pipeline resulted in identifying more long contigs, with an average of maximum contig lengths across samples of 51,312 bp (maximum 108,191 bp for sample BBC) with an average of the median contig lengths of 6,713 bp (maximum 16,828 for sample FHC). The sample WHC had notably shorter contigs than others (maximum 4,552 bp, median 807 bp).

### Candidate phage detected with family-level and genus-level abundances

**Figure 2:**
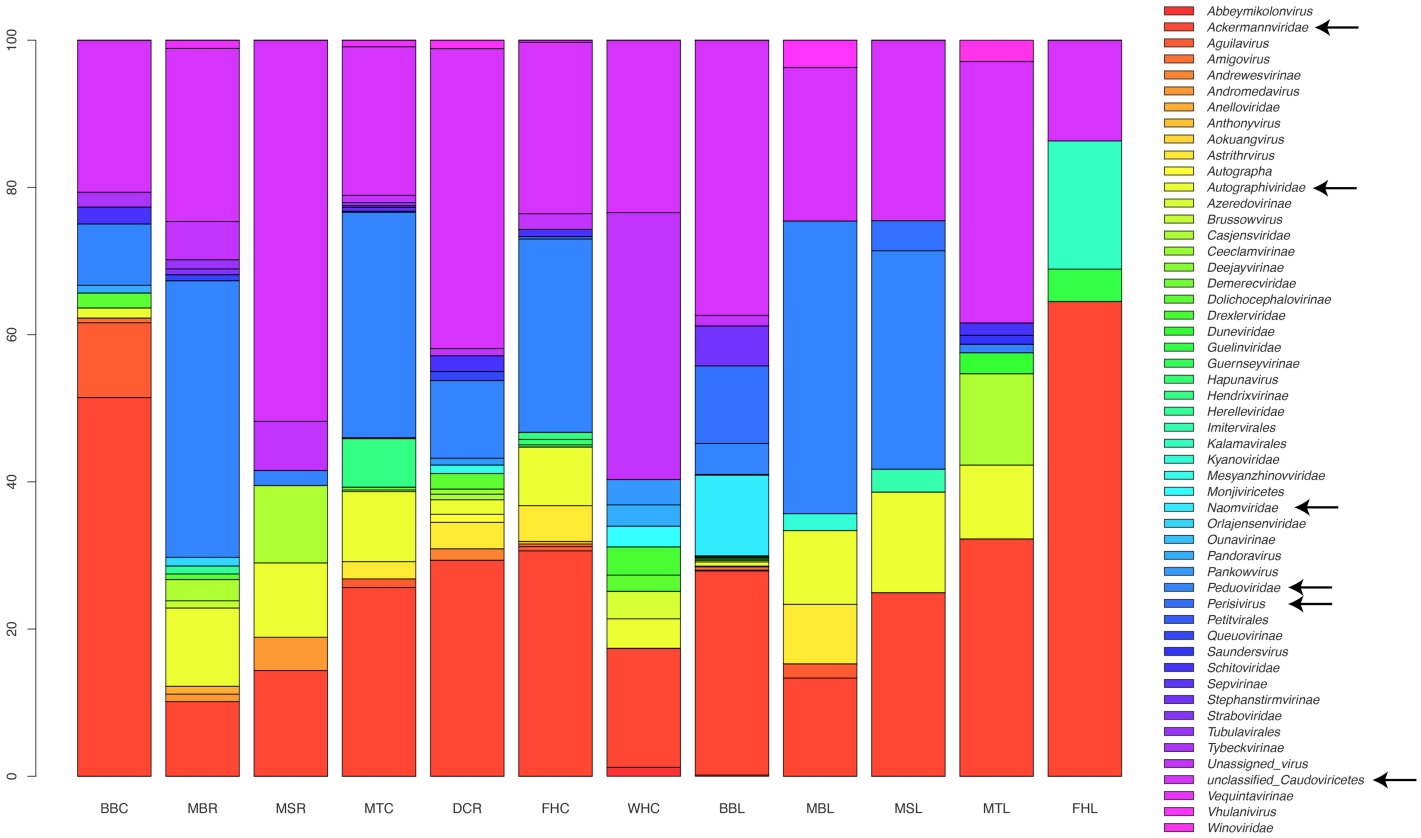
Relative abundance of putative phages detected from microbiomes of *Musa* genotypes and tissues, classified to the family level using GDV Tool, calculated after normalizing read coverage among samples. For ease of visualization, arrows are used to point to the most abundant groups, including unclassified Caudoviricetes, and the five most abundant virus families across samples. Samples on the x-axis: BBC and BBL = *Musa balbisiana* Thai Black corm and leaf, DCR and DCL = Dwarf Cavendish root, and leaf, FHC and FHL = FHIA-25 corm and leaf, MBR and MBL = *Musa balbisiana* root and leaf, MSR and MSL = *Musa sikkimensis* root and leaf, MTC and MTL = *Musa textilis* corm and leaf, WHC and WHL = Williams Hybrid corm and leaf.

After normalizing for relative numbers of reads, phage abundances at the family level varied among sampled genotypes and tissues (Figure 2). Predicted phages from the GDV pipeline were classified to 51 families, with the most common classification, at ~30% abundance, being “unclassified Caudoviricetes.” The next most abundant group was family *Ackermannviridae* (~27% abundance), followed by *Peduoviridae* (15.7%), and *Autographiviridae*, *Perisivirus*, *Naomviridae*, *Stephanstirmvirinae*, and *Casjensviridae* (at 6%, 4.2%, 3.9%, 2%, and 1.7% abundances, respectively). Among *Musa* genotypes and tissues, the pattern of phage abundance at the family level was variable (Figure 2), with Williams Hybrid roots (WHC) having a larger proportion of unassigned viruses than the others, FHIA-25 Hybrid leaves (FHL) having a larger portion of *Kalamavirales*, *M. balbisiana* Thai Black roots (BBC) having a larger portion of *Aguilavirus*, and MBR, MSR, and MTL having larger proportions of *Casjensviridae*.

At the genus-level, predicted phage presence/absence and abundances from the GDV pipeline differed among samples, with most phage genera being unique to each sample. Overall, the most abundant phage genera were *Rhizobium phage*, *Vhulanivirus*, *Salmonella phage*, *Brucella phage*, *Noahvirus*, *Rhodovulum phage*, *Eganvirus*, *Peduovirus*, and *Nanhaivirus*. No genera were universally shared among all samples, but those most commonly found across samples included *Salmonella phage*, *Rhizobium phage*, *Pseudomonas phage*, *Ochrobactrum phage*, and *Rhodovulum phage*. In roots, the most abundant phages were *Salmonella phage*, *Rhizobium phage*, *Peduovirus*, *Quadragintavirus*, *Yersinia phage*, *Brunovirus*, *Eganvirus*, and *Klebsiella phage*. In leaves, the most abundant phages were *Rhizobium phage*, *Vhulanivirus*, *Brucella phage*, and *Noahvirus*. Overall phage taxa identification and abundances were similar for the blastn and GDV approaches except that the blastn method failed to detect about one- to two-thirds of the phages detected with GDV (Table 2; Supplementary Table 1).

**Figure 3:**
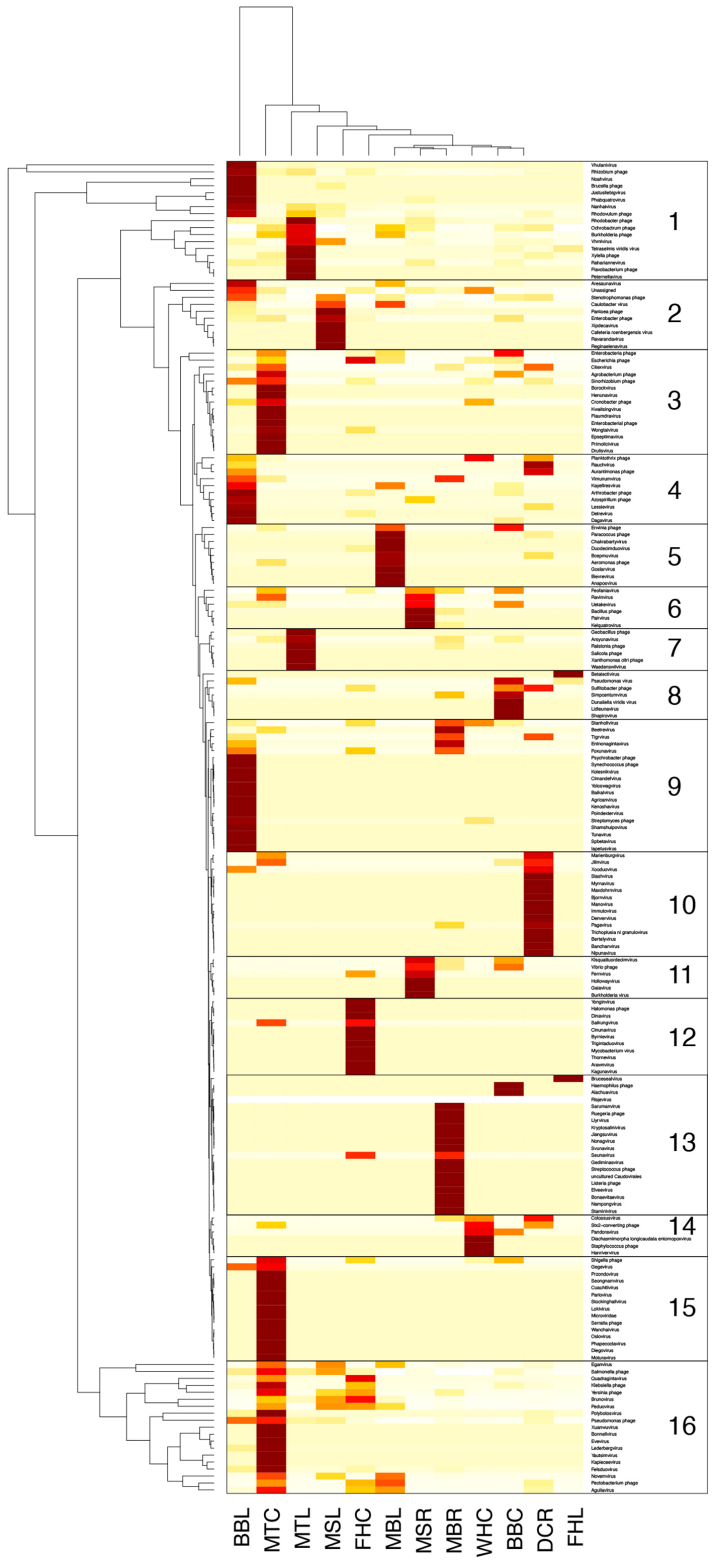
Relative abundance of putative phages detected from microbiomes of *Musa* samples classified to the genus level using GDV Tool, calculated after normalizing read coverage among samples. Heatmap has been scaled across rows and clustered by similar rows to reveal similarities in abundance patterns among sets of phage and host plant. Heatmap color: light to dark represents lowest to highest abundance. Boxes enclose 16 predominant abundance clusters. Samples on the x-axis: BBC and BBL = *Musa balbisiana* Thai Black corm and leaf, DCR and DCL = Dwarf Cavendish root, and leaf, FHC and FHL = FHIA-25 corm and leaf, MBR and MBL = *Musa balbisiana* root and leaf, MSR and MSL = *Musa sikkimensis* root and leaf, MTC and MTL = *Musa textilis* corm and leaf.

Phage genera abundances plotted as a clustered heatmap scaled across rows (Figure 3) showed sets of 6 to 20 phages (numbered boxes 1 to 16 in Figure 3) which clustered together with high abundance (columns of dark boxes in heatmap). These sets of phages at higher abundance showed limited overlap across genotypes and tissues, i.e., most samples had unique patterns in highly abundant phages.

### Shared and unique phage communities

**Figure 4:**
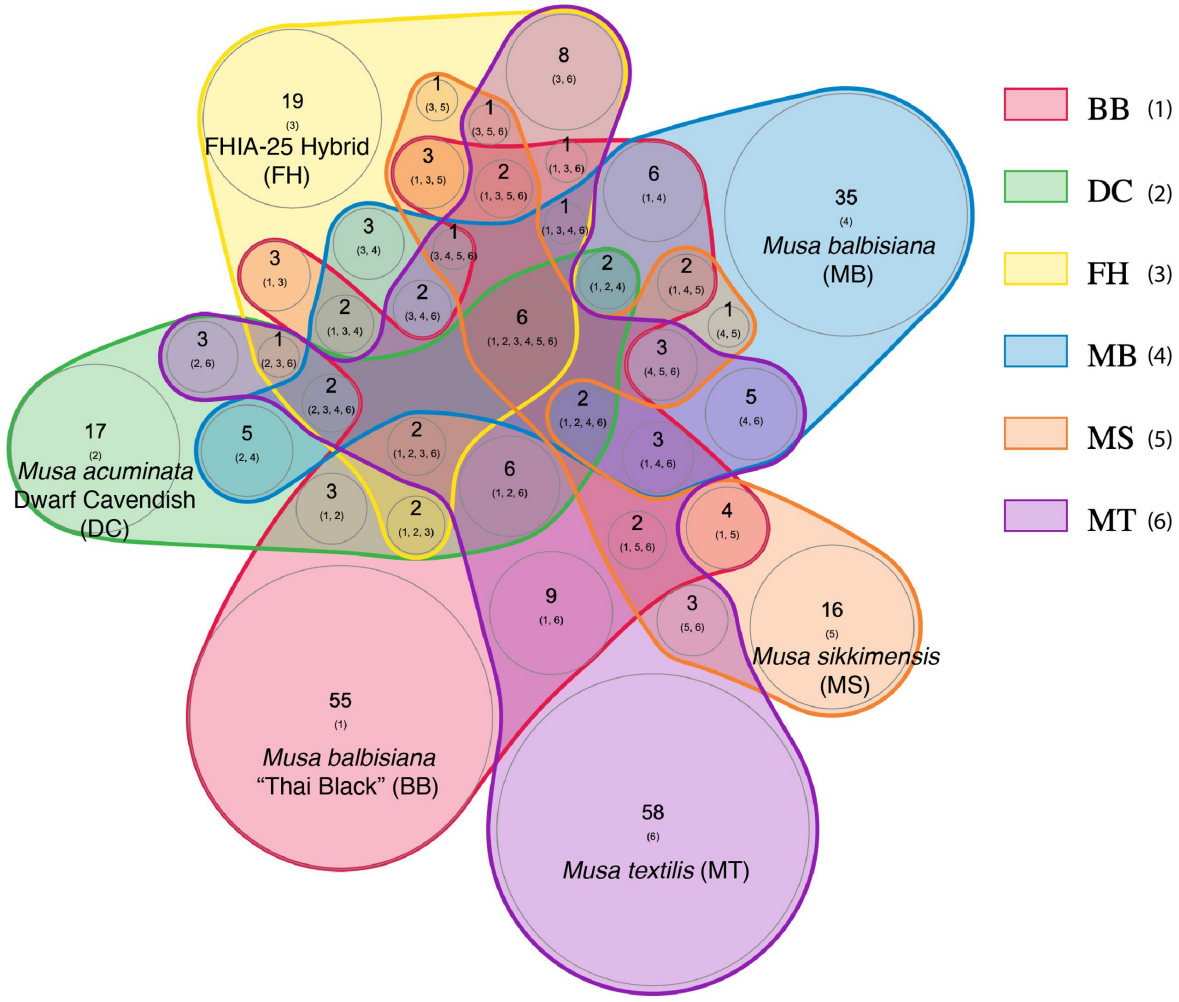
Putative phage community overlap at the lowest taxonomic-levels (species or isolates) within the endosphere microbiomes of 6 *Musa* genotypes, combining leaf and root tissues for each genotype, shown as a proportional Venn diagram, with each *Musa* genotype depicted with a separate color. Numbers in circles indicate numbers of shared phages between *Musa* genotypes or phages unique to a genotype, while numbers in parentheses within circles indicate numbers of shared or unshared orthologous genes among phages in each circle.

Comparing predicted phages at the lowest taxonomic level (species or isolate) across *Musa* genotypes, showed that most predicted phages were unique to each plant genotype with only a few occurring in more than one plant host (Figure 4). The specific predicted phages that were universal to all sampled *Musa* genotypes were *Rhodovulum phage* RS1, *Stenotrophomonas phage* S1, *Ochrobactrum phage* POA1180, *Brunovirus* SEN34, *Salmonella phage* 118,970 sal3, and unassigned phages, whereas predicted phages that were nearly universal among *Musa* genotypes included *Rhizobium phage* RR1-B, *Rhizobium phage* RR1-A, *Rhizobium phage* 16–3, *Pectobacterium phage* ZF40, *Felsduovirus* RE2010, *Aguilavirus* mEp043, *Vhulanivirus* Shpa, *Nanhaivirus* D5C, *Eganvirus* PsP3, and *Yersinia phage* P37. There were 36 phages that were uniquely shared among sets of diploid genotypes (BB, MB, MT, MS), but we did not detect any uniquely shared phages among triploids (FH, DC; Supplementary Table 1).

Diversity analysis showed that diploid *Musa* plants (genotypes BB and other diploids) had higher phage alpha diversity compared to triploid (domesticated) *Musa* plants (genotypes AAA and AAB): Shannon diversity (*p* < 0.01, Hutcheson *t*-test) ranging from 4.9 (diploid plants) to 4.5 (triploid plants). Furthermore, statistical analyses showed that root tissues had significantly higher alpha diversity than leaf tissues: Shannon diversity (*p* < 0.01, Hutcheson *t*-test) ranging from 6.5 (root samples) to 4.4 (leaf samples).

### Phage gene repertoire and protein family domain abundance

**Figure 5:**
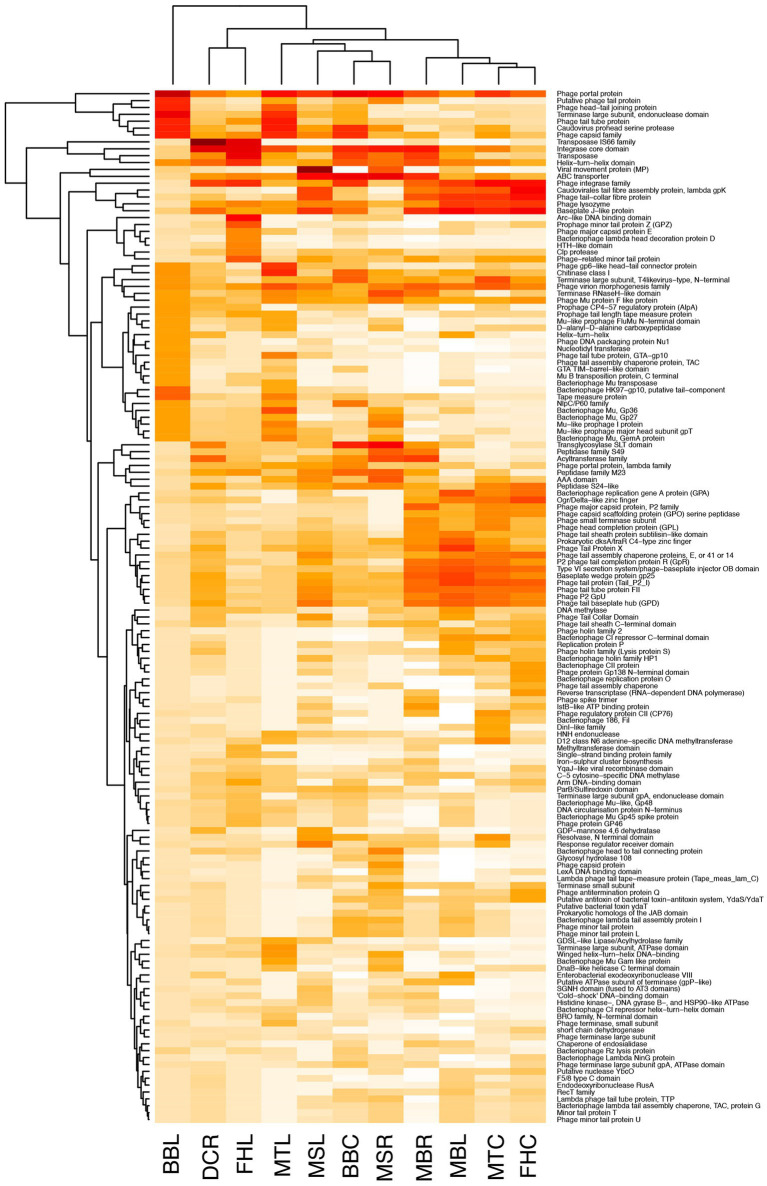
Relative abundances of predicted phage protein domains (pfams) for the 150 most abundant pfams detected from *Musa* samples microbiomes using the DRAMv software. Heatmap color: light to dark represents lowest to highest abundance. Samples on the x-axis: BBC and BBL = *Musa balbisiana* Thai Black corm and leaf, DCR and DCL = Dwarf Cavendish root, and leaf, FHC and FHL = FHIA-25 corm and leaf, MBR and MBL = *Musa balbisiana* root and leaf, MSR and MSL = *Musa sikkimensis* root and leaf, MTC and MTL = *Musa textilis* corm and leaf.

Using the less-sensitive blastn pipeline and annotating genes with prokka, among contigs with strong blast similarity to viruses, many (52.7%) contained no predicted genes, either because candidate open reading frames did not pass similarity filters of the prokka software or because contigs were too short (e.g., 63.8% of contigs were <1,000 bp). Nevertheless, prokka annotation resulted in 887 predicted genes among these contigs. Of these, 733 (82.6%) were annotated as “hypothetical protein.” Phage gene orthologs, analyzed in Roary, showed a similar pattern, with most orthologous gene clusters being unique to individual *Musa* genotypes. Using the contigs identified with the VirSorter2-vConTACT2-GDV pipeline, far more contigs could be annotated with virus genes or virus-like protein coding motifs (pfam) using the DRAMv software (Supplementary Table 4), resulting in 1,038 unique pfam domains across samples with ~400 to 600 unique viral pfams in most samples. There were up to 1,901 contigs per sample with predicted viral pfam domains. The most abundant of these genes or pfam domains, normalizing among samples and accounting for read coverage were: phage portal protein, Integrase core domain, Phage integrase family, Baseplate J-like protein, ABC transporter, Helix-turn-helix domain, Phage capsid family, Phage virion morphogenesis family, Transposase, and Caudovirus prohead serine protease. Comparing relative abundances of phage pfam domains across samples (Figure 5) showed no particular pattern across *Musa* genotypes or tissue types; however, assemblages of pfams did show some clustering within samples (e.g., MBR, MBL, MTC, and FHC had numerous abundant phage tail proteins in common, compared with other samples).

### Synteny and phylogenetic analysis of several predicted phages

**Figure 6:**
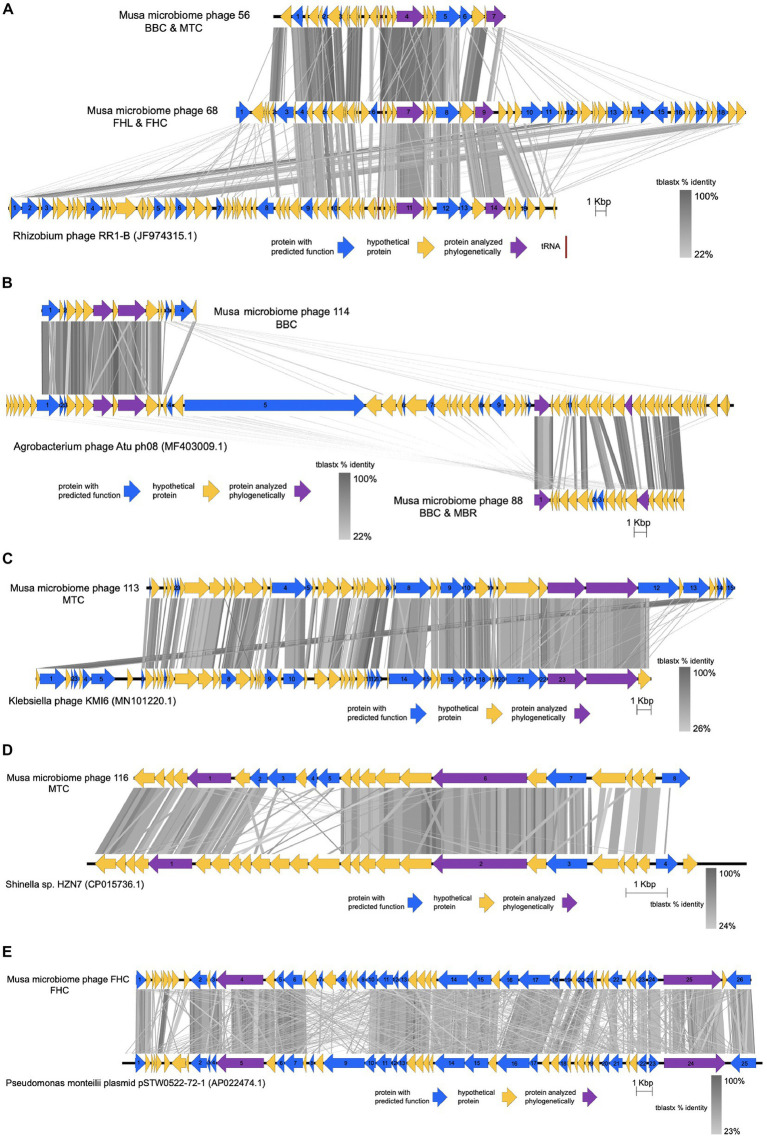
Annotated PHASTER-predicted phages or prophages from *Musa* microbiomes compared to similar full-length reference sequences from GenBank, showing synteny and tblastx similarity (in grayscale bands) between predicted proteins of known (blue arrow) or unknown (yellow arrow) function, showing proteins that were analyzed phylogenetically (purple arrow) in Supplementary Figures 1–15. **(A)** Predicted phages 56 and 68 aligned to *Rhizobium* phage RR1-B. **(B)** Predicted phages 114 and phage 88 aligned to *Agrobacterium* phage Atu ph08. **(C)** Predicted phage 113 aligned to *Klebsiella* phage KMI6. **(D)** Predicted phage 116 aligned to *Shinella* sp. phage HZN7. **(E)** Predicted “FHC phage” aligned to *Pseudomonas monteillii* plasmid pSTW0522-72-1.

To assess predicted phage synteny for several of the most common and well-characterized phages, 8 predicted phage or prophage regions detected by both the GDV pipeline and PHASTER were analyzed compared to references in a gene-by-gene manner and with phylogenetics. These predicted phages ranged from 12.1 to 49.3 kb, with 16 to 56 predicted genes, with two of these phage regions classified as “intact,” 4 as “incomplete,” and 2 as “questionable” in PHASTER. Comparative synteny analysis with closest reference sequences from GenBank is shown in Figure 6 and phylogenetic analysis of the two longest predicted genes from each of these 8 phages is shown in Supplementary Figures 1–15.

Intact predicted “Phage 56” from samples BBC and MTC, and incomplete “Phage 68” from samples FHL and FHC were most similar to *Rhizobium* phage RR1-B (NCBI accession JF74315.1; Figure 6A), with moderately high synteny conservation, and higher blastx identity to one another than to the reference sequence. Notable genes present in these *Musa* microbiome phages that were not present in the reference phage (Supplementary Table 5) include genes encoding an ISL3 family transposase, a partial Shiga-like toxin 2 subunit A (63 amino acids long, with 66% identity to prophage Shiga-like toxin sequences integrated into *Agrobacterium* species), and a holin. Phylogenetic analysis of the DNA methyltransferase/methylase N-4/N-6 gene showed these predicted phages to be most closely related to *Rhizobium skierniewicense* and *Agrobacterium* sp. AGB01 in a well-supported clade with other *Agrobacterium*/*Rhizobium* spp. Supplementary Figure 1, which was nested between clades comprised *Agrobacterium vitis* and *Agrobacterium leguminosarum*. Phylogenetic analysis of the “Phage 68” C-5 cytosine specific DNA methylase gene showed a similar pattern to that of the DNA methytransferase gene described above, whereas the “Phage 56” DNA primase gene clustered with *Rhizobium straminoryzae* phage/plasmid sequences and other *Rhizobium* spp. (Supplementary Figures 2, 3).

Incomplete predicted “Phage 114” from sample BBC and “Phage 88” from samples BBC and MBR were most similar to the flanking ends of *Agrobacterium* phage Atu ph08 (NCBI accession MF403009.1), with relatively high synteny (Figure 6B). Notable genes present in these phages that were absent in the reference phage (Supplementary Table 5) include a DNA adenine methyltransferase gene, a CzcR-like response regulator gene, and an endolysin gene. Phylogenetic analysis showed a ‘Phage 88’ gene annotated as encoding an integrase arm-type DNA binding domain protein clustered closely with an undescribed Proteobacteria bacterium within a clade of *Agrobacterium tumefaciens* (*=A. radiobacter*) complex while its gene for a UvrB/UvrC motif containing protein clustered similarly (Supplementary Figures 4, 5). Similarly, genes from “Phage 114” annotated as hypothetical proteins clustered with *A. tumefaciens* and other *Agrobacterium*, including *Agrobacterium salinitolerans* (Supplementary Figures 8, 9).

A very long phage-like region denoted as “questionable,” denoted here as “Phage 113” from sample MTC, had high gene conservation and synteny conservation to *Klebsiella* phage KMI6 (NCBI accession MN101220.1; Figure 6C). There were few genes that differed substantially between this phage and the reference, except annotated gene 12 in “Phage 113” (Supplementary Table 5) encoding a putative tail fiber protein which appeared to be absent in the reference. Phylogenetic analysis of two putative internal core protein genes indicated clustering with a clade containing a variety of *Klebsiella* phages, *Escherichia* phages, and undescribed *Caudovirales* sp. with high bootstrap support but large sequence divergence (i.e., long branch lengths; Supplementary Figures 6, 7).

Incomplete “Phage 116,” from sample MTC, was most similar to a prophage of *Shinella* sp. HZN7 (NCBI accession CP015736.1), but with a middle segment of uniquely missing and different genes and some regions with highly diverged (low blastx identity) genes (Figure 6D). Genes present in this phage that were absent in the reference (Supplementary Table 5) included a conserved transposable phage protein gene, a host-nuclease inhibitor protein “gam” gene, and a putative DNA ends-protecting protein. Phylogenetic analysis of a gene for a peptidoglycan-binding domain protein and a gene for a transposase C-terminal domain-containing protein showed this phage to be closest to *Shinella* sp. phage HZN7, with very weak bootstrap support for various sister clades (Supplementary Figures 10, 11).

Intact “Phage FHC” from sample FHC, which assembled as one complete contig, was most similar to *Pseudomonas monteillii* plasmid pSTW0522-72-1 (NCBI accession AP022474.1) with very high gene content similarity (Figure 6E). Phylogenetic analysis of the predicted phage tail tape measure protein gene and a hypothetical protein gene showed a strongly supported cluster with *Pseudomonas* sp. WS 5027, within clades containing *P. monteillii*, *Pseudomonas aeruginosa*, and *Pseudomonas alloputida* (Supplementary Figures 14, 15).

Finally, predicted “Phage DCR,” which was assembled as one large contig and was denoted as “questionable,” had no significantly similar full-length reference match, and so could not be analyzed with Easyfig. Nevertheless, this predicted phage had 17 of 19 predicted genes matching phage-like proteins. Phylogenetic analyses of genes for a phage tail tape measure protein and a head maturation protease showed strong bootstrap support for clustering with *A. tumefaciens* (=*A. radiobacter*) and *Rhizobium tropici*, and *Rhizobium metallidurans* and *Rhizobium pusense*, respectively. However, the branch lengths were quite long, suggesting significant sequence divergence in both cases (Supplementary Figures 12, 13).

### Plant DNA viruses or endogenous viral elements detected

**Figure 7:**
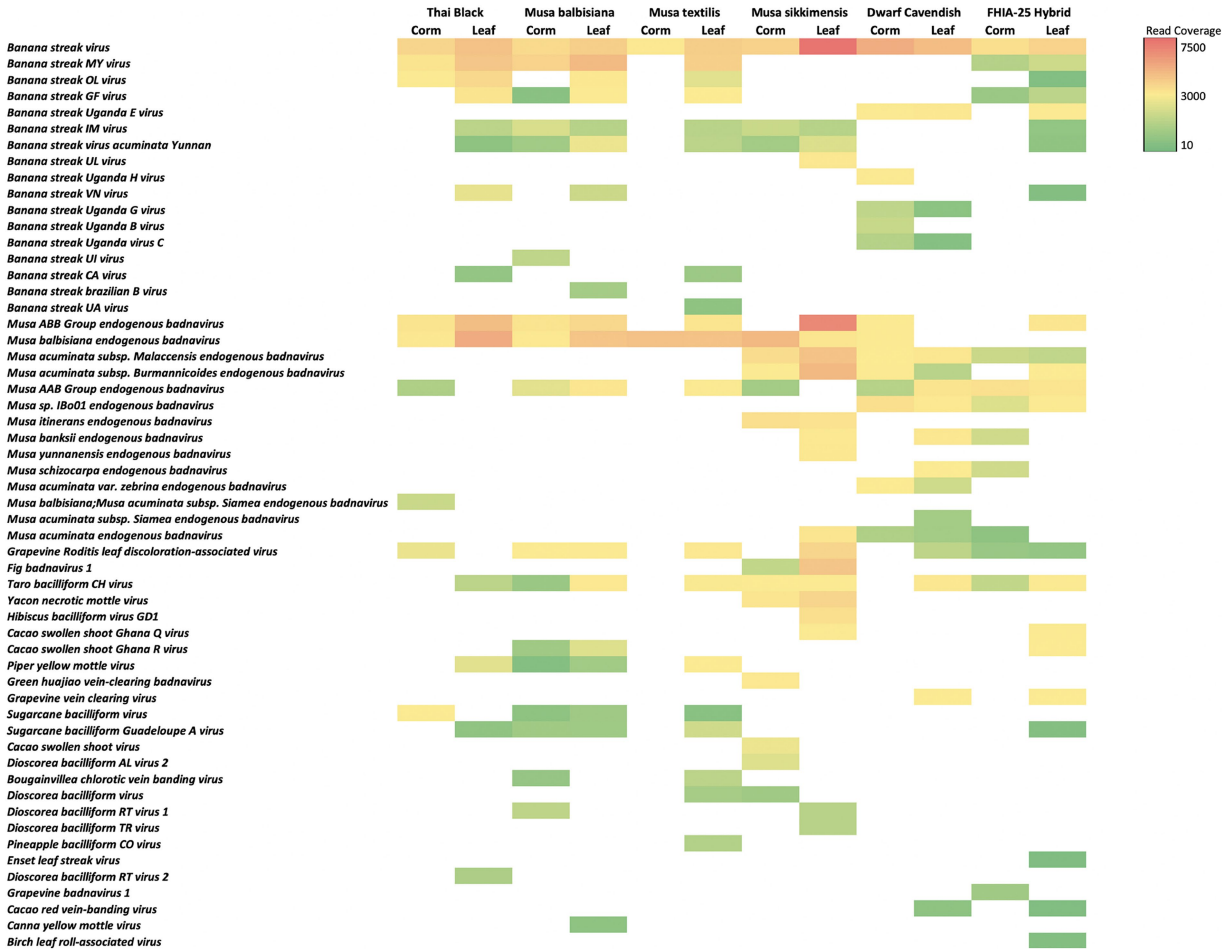
Abundance of putative endogenous viruses in 12 *Musa* samples from leaf and corm tissues, based on blastn hits to assembly contigs shown as a heatmap of coverage, shown in color legend, after normalization to control for differences in reads across samples.

The contigs matching endogenous viruses included both short and long contigs, many of which had high blastn similarity to references in NCBI databases (Supplementary Table 6). For example, the maximum length of a contig that matched endogenous viruses was 37,479 bp, whereas 41 of these endogenous virus-like contigs were >3,000 bp, 41.6% of contigs were >900 bp, and 90% of contigs were >300 bp. Blast hit regions were relatively long, with 81.9% of hits being >300 bp and 112 hits being >900 bp. Nucleotide identities for hit regions were variable, with 55.5% of hit regions being >95% identity and 34% of hits being >98% identity. Among matches to reference endogenous viruses annotated as “complete,” 39% of the matching contigs showed 100% coverage (i.e., indicating the contig included the complete endogenous virus genome; see Supplementary Table 6, which includes sequences) and inspection of predicted ORFs in Geneious showed full, intact predicted genes. The sum of read coverage of predicted endogenous viruses (Table 2) was quite high (40,481 X) despite low levels of banana genomic DNA in these enriched microbiome layers (Figure 1). Endogenous virus-like reads, calculated across all banana genotypes (Figure 7; Supplementary Table 7), were mostly from the order Ortervirales, with most matches to family Caulimoviridae, in the genus *Badnavirus*. Among *Badnavirus* matches, 56 species were identified. In total, 42.13% of reads matched the broad group of badnaviruses denoted endogenous banana streak viruses (eBSVs). Seventeen distinct species of eBSVs were identified from across sampled *Musa* genotypes. The most abundant *Badnavirus* species were *Banana streak virus* (BSV; 60.97% of eBSVs reads) and *Banana streak MY virus* (BSMYV; 22.42% of eBSVs reads). Other eBSVs were detected at less than 5% read coverage. Additionally, 14 species matched other *Musa*-group endogenous badnaviruses (Figure 6; Supplementary Table 7).

Other non-*Musa* plant viruses detected include 26 additional *Badnavirus* species, including several that were present in multiple samples, such as *Grapevine Roditis leaf discoloration-associated virus*, *Taro bacilliform CH virus*, *Piper yellow mottle virus*, *Sugarcane bacilliform virus*, and *Sugarcane bacilliform Guadeloupe A virus*.

### Shared and unique viral species from banana endogenous communities

Analyses of predicted endogenous plant virus diversity among samples showed that only *Banana streak virus* was universal to all root and leaf samples for all *Musa* genotypes. Overall, 71.68% of endogenous plant virus reads were from leaf samples, from which 17 species were unique to leaf tissues, whereas 9 species were unique to root samples. Comparing *Musa* genotypes, 17 endogenous viruses were unique to Thai Black roots (BBC), while 3 viruses were unique to *M. balbisiana* roots (MBR). Among BB genotypes (MBR and BBC) only 3 viruses where shared. Also, 14 viruses were unique to *M. textilis* roots (MTC), 3 viruses were unique to *Musa sikkimensis* root (MSR), 6 viruses were unique to Dwarf Cavendish roots (DCR; AAA genotype), and 4 viruses unique to FHIA-25 Hybrid roots (FHC; AAB genotype; Figure 7; Supplementary Table 7).

Several candidate viruses were exclusively detected from diploid (wild) genotypes (BB/MB, *M. textilis*, and *M. sikkimensis*; Figure 7): *Banana streak Brazilian B virus*, *Banana streak CA virus*, *Banana streak UA virus*, *Banana streak UI virus*, and *Banana streak UL virus*. Some viruses were exclusively detected from diploids and FHIA-25 (AAB genotype) samples: *Banana streak GF virus*, *Banana streak IM virus*, *Banana streak MY virus*, *Banana streak OL virus*, *Banana streak virus acuminata Yunnan*, and *Banana streak VN virus*. Other viruses were found exclusively in Dwarf Cavendish (AAA genotype): *Banana streak Uganda B virus*, *Banana streak Uganda E virus*, *Banana streak Uganda G virus*, *Banana streak Uganda H virus*, and *Banana streak Uganda virus C*.

Diversity analyses of candidate endogenous viruses showed that diploid (wild) plants had higher alpha diversity compared to triploid domesticated (AAA and AAB) plants: Shannon diversity (*p* < 0.01, Hutcheson *t*-test) ranged from 3.32 for triploid plants to 4.02 for diploid plants. Furthermore, statistical analyses showed that leaf tissues had higher alpha diversity than root tissues. Shannon diversity (*p* < 0.01, Hutcheson *t*-test) ranged from 3.9 (leaf samples) to 3.4 (root samples). The same pattern and similar statistically significant differences were found after removing *Banana streak virus* which occurs in every plant and tissue and is therefore more likely incorporated into the banana genome.

## Discussion

We compared endophytic virus-like sequences from sympatric banana plants including wild diploid and domesticated triploid varieties as a critical first step toward assessing how these viromes may impact microbiomes. Results revealed numerous novels or highly divergent predicted phages and dsDNA viruses or endogenous viral elements (EVEs) with generally non-overlapping community structures despite the shared environments of these plants, suggesting hosts drive the composition of these DNA virus communities.

Comparing phage communities between banana genotypes demonstrated clear differences at the phage family-, genus-, and species/isolate-levels, suggesting an influence of plant host history (wild diploid vs. domesticated triploid), as expected given previous studies of domesticated vs. wild relative microbiomes (Liu et al., 2017; Mertens et al., 2021; Nakkeeran et al., 2021). The lack of overall lower-level taxonomic overlap among *Musa* plants grown together in the small-scale farm suggests that these endophytic phages either do not easily transfer among plants or that plant hosts control or limit their communities. Furthermore, data showed a pattern of phage communities unique to each plant (shown in Figure 3) suggesting possible “phage consortia” may be able to infect or persist within these endospheres. If these distinct endosphere phageomes persist in healthy plants over the long term, presumably actively infecting endophytic bacteria. Despite our efforts to functionally annotate these phage communities and the great diversity of predicted pfam domains, the resulting lists of pfams provided limited insight: most pfams represent standard viral components. However, and the clustering of several samples (*M. balbisiana*, *M. textilis*, and FHIA-25 Hybrid) in their abundance profiles for many phage tail components raises interest in the possible activity of these phage. Further work will be essential to explore these possibilities as part of a broader investigation of the impact of endophytic phages on the plant microbiome’s contribution to disease resistance.

Plant tissue and genotype influenced the composition of predicted phage communities, as expected based on studies of bacterial endophyte communities (Lundberg et al., 2012; Liu et al., 2017; Maheshwari and Annapurna, 2017; Fitzpatrick et al., 2018; Afzal et al., 2019; Mertens et al., 2021; Nakkeeran et al., 2021; Wippel et al., 2021). For example, even though there were higher relative levels of viruses in leaves than roots, these virus blast hits were predominantly endogenous plant viruses, with few phage or prophage hits. This result is consistent with roots being primary sites of bacterial colonization, with leaves hosting a subset of root-colonizing microbiota (Fitzpatrick et al., 2018; Wippel et al., 2021). However, several predicted phage phages appeared to be unique to leaf tissues, for example, *Burkholderia* phage DC1. Its host, members of the *Burkholderia cepacia* complex, are important intracellular endophytes that are often integral to plants, conferring key protective benefits (Pan et al., 1997; Mendes et al., 2007; Mercado-Blanco and Lugtenberg, 2014).

In the endophytic microbiome of *Musa* spp., we found predicted phage or prophage sequences that were diverged from those of reference phage, suggesting that these phages may be unique to bacterial endophytes or pathogens of banana. Among the abundant and widely shared phages identified in these *Musa* samples, several resembled *Rhizobium* phage RR1-A and RR1-B, which are temperate phages first characterized from deep subseafloor sediments (Engelhardt et al., 2013), but also found associated with the rhizosphere of plants, such as *Agave americana* (Ruíz-Valdiviezo et al., 2017). Phylogenetic comparisons suggested that this phage may be related to phage infecting *R. skierniewicense*, an endophytic tumor-causing rhizobium (Pulawska et al., 2012). We found two diverged variants of this phage, one in diploids *M. balbisiana* and *M. textilis* (denoted “Phage 56”) and the other in the triploid FHIA-25 Hybrid (denoted “Phage 68”), with higher sequence similarity between these contigs than to the reference, suggesting possible *Musa*-specific variants and possible phylodiversification of this phage or its bacterial host. Detection of such RR1 “predatory” lysogenic phages (Engelhardt et al., 2013) in *Musa* spp. combined with our detection of an additional a holin gene and a partial Shiga-like toxin gene in these phages raises interest in their capacity to regulate Rhizobiaceae in the endophytic microbiome.

Another widely observed group of predicted phage in these *Musa* samples matched *Klebsiella* phage, targeting *Klebsiella* bacteria which are a focus for their plant growth promoting properties. Although initial blast similarity suggested the numerous *Klebsiella* phages in these *Musa* microbiomes were most similar to prophage in clinically important *Klebsiella pneumoniae* (Bleriot et al., 2020), the sequences were quite diverged from known clinical and agricultural strains in GenBank, likely reflecting distinct phage variants. Diverse *Klebsiella* strains including strains of *K. pneumoniae* are nitrogen-fixing diazotrophs which colonize plants well, especially monocots (Dong et al., 2003). Many strains promote plant growth and some have been shown to be protective against diseases such as Fusarium wilt in banana (Nakkeeran et al., 2021). Together, these findings raise interest in the novel *Klebsiella* phage variants found in this study. As with the RR1-like sequences, the divergence among *Klebsiella* phage-like sequences among our sampled *Musa* plants suggest *Musa*-specific variants and possible phylodiversification in these hosts.

A predicted phage found only in the *M. textilis* microbiome distantly matched a phage from the nicotine-degrading alphaproteobacterial strain *Shinella* sp. HZN7 (Ma et al., 2014). *Shinella* species have been found as endophytes in several studies and are remarkable for their diverse biosynthetic properties supported by large genomes (~7.35 Mbp), including electroproducing endophytes (Qiu et al., 2016; Ling et al., 2022). The additional transposable phage protein gene detected here may suggest this is a transposable phage with the ability to rearrange host genomes (Toussaint and Rice, 2017), while the other extra genes (a host-nuclease inhibitor protein ‘gam’ gene and a DNA ends protecting protein) which protect the phage against host RecBCD-driven degradation suggest this could be a formidable regulator of its host endophytes.

*Pseudomonas* spp. are common in endophytic microbiomes, conferring a wide range of plant-beneficial traits (Nakkeeran et al., 2021), hence, our discovery of an intact phage-like region matching a plasmid from *Pseudomonas monteillii* (an environmental and human pathogenic species) or *Pseudomonas* sp. WS 5027 (a psychrophilic species initially found in milk; Maier et al., 2020) was of interest. Curiously, this predicted phage/plasmid was only found in the FHIA-25 Hybrid banana, suggesting specificity of this phage’s host. We also detected at lower coverage, a *Pseudomonas fluorescens* phage KNP-like sequence. This sequence matched a cluster of phages of interest for their potentially important role in regulating plant-specific *P. fluorescens* (Nowicki et al., 2017).

The discovery of two fragments matching phage Atu_ph08 from the plant pathogen *Agrobacterium tumefaciens* (=*A. radiobacter*) in Thai Black *M. balbisiana* is of interest due to recent evidence suggesting that these novel T7-like phages may be lysogenic and could regulate pathogenic *Agrobacterium* (Attai and Brown, 2019). However, our seqeunces from banana lacked the large helicase gene found in the Atu_ph08 phage: it is unclear whether this indicates assembly artifacts due to repeat elements or diversity among metagenomic variants, or whether these prophage regions have lost the helicase and are degrading. Another candidate *A. radiobacter* phage, “Phage DCR” from Dwarf Cavendish, was too diverged from reference sequences in GenBank to comparatively analyze gene synteny, and may represent another novel host-specific virus or prophage.

We also found predicted phages with potential to regulate *Erwinia*-, *Pectobacterium*-, and *Ralstonia*-associated diseases which have been reported as the most devastating bacterial diseases of banana worldwide, after *Xanthomonas* wilt (Blomme et al., 2017). We found a match to *Erwinia* phage EtG, which clusters with Enterobacteriales phages of wide host ranges (Thompson et al., 2019), and a match to the *Pectobacterium* phage ZF40, hosts of which cause Enterobacteraceae derived soft rot diseases (Czajkowski, 2015; Comeau et al., 2017). We also found low levels of *Ralstonia* phage in *M. sikkimensis* and Thai Black roots, which is important given previous studies that showed phages isolated from banana soils may control *R. solanacearum*, causing devastating banana Moko disease (Ramírez et al., 2020) and similarly, phages appear effective in controlling *Ralstonia syzygii* sub sp. *celebesensis (Rsc)*, causing banana blood disease (Murthi et al., 2021).

In particular, our results showing a large proportion of the reads from sequencing the bacterial microbiome layer were unclassified even at kingdom level supports the hypothesis that there may be many undescribed DNA viruses in *Musa* tissues. Consistent with this result, the most abundant phages were “uncharacterized Caudoviricetes” and a majority of blast-detected phage-like regions could not be annotated to known genes, although GDV performed somewhat better at function prediction than the simple prokka/DIAMOND approaches. Furthermore, we suspect that our Nycodenz enrichment protocol, which was developed for isolating bacteria may not be optimal for phage particles, some of which we speculate may be lost in the pellet, further reducing the measured abundance and diversity of phage in our study. Together, these arguments all point to hidden undiscovered phage diversity in the *Musa* endosphere. This is on the one hand surprising, given the challenges of phage transmission one might imagine within the confines of plant tissues. Although metagenomic virus detection workflows are improving (Bin Jang et al., 2019; Roux et al., 2019; Vilsker et al., 2019; Pratama et al., 2021; Turner et al., 2021), MAG approaches are still less often used for detecting phage communities compared to bacterial communities, and sequence databases for phage genes still lag behind that of bacterial genes (Dion et al., 2020). Although we found the VirSorter2-vConTACT2-GDV pipeline to be several times more sensitive at detective viruses than our initial blastn-based pipeline, we note that vConTACT2 was somewhat poorer at taxonomic classification than GDV, as has been reported by others (Zhu et al., 2022). This performance difference is likely due to an abundance of short contigs in our samples.

Although detection of dsDNA plant viruses was not the primary goal of this study, given the methods used to enrich microbial cells, we found a surprising proportion of reads (e.g., at a total of >40,000 × coverage) and assembled contigs (e.g., 708 distinct taxa) matched plant DNA viruses, including both predicted intact plant viruses and fragmented or degrading viral fragments. This result was more surprising, given the tiny relative number of copies of *Musa* genomic DNA in our data. Unlike the diversity of phage, which was higher in root tissues, plant viruses were richer in leaves, and unlike the largely non-overlapping phage communities among genotypes, abundant plant viruses tended to be shared across all tissues and *Musa* genotypes. This is likely because these DNA plant viruses, such as the banana streak viruses, largely occur as endogenous, replicating forms universally integrated into *Musa* genomes (Geering et al., 2005; Gayral et al., 2008). The high coverage and abundance of these in our data, despite limited host DNA levels, is consistent with their high copy number in the host DNA. In addition, we found differences among genotypes: diploids had more diverse DNA plant virus communities than triploids, with many unshared viruses, including numerous non-streak and non-*Musa* viruses, suggesting a mixture of resident and transient DNA viruses in these samples. These findings are important because cultivars of commercial banana (triploid *M. acuminata*) are susceptible to infection by a broad range of badnaviruses (Harper et al., 2002; Geering et al., 2005), impacting breeding programs as viral DNA integrated into the nuclear genome of *M. balbisiana* (BB genotype) spreads (Geering et al., 2005) leading to systemic infection when the plants are stressed (Muller et al., 2021). Insertion of a badnavirus promoter next to an endogenous plant gene may change transcription levels and alter tissue specificity of expression and give rise to infection (Matzke et al., 2004). Yet, most EVEs are thought to be “viral molecular fossils” in wild plant (Jones et al., 1999), with some latent plant viruses potentially providing a benefit to wild host plants (Pagán et al., 2012; Tripathi et al., 2019). Given our findings of differences in EVEs and other relatively abundant non-streak disease-causing dsDNA viruses in these *Musa* plants (e.g., Grapevine Roditis leaf discoloration-associated virus, Fig badnavirus 1, Taro bacilliform CH virus, Yacon necrotic mottle virus, and Dioscorea bacilliform RT virus), we propose future focused work to improve enrichment protocols, including target capture, to economically assay the diversity of these plant DNA viruses.

In conclusion, our results contribute to the small but growing collection of studies that characterize DNA virus communities within plants, a first step toward examining how endophytic viruses may regulate plant microbiomes. While we found several predicted viruses with similarity to known pathogens, most predicted virus-like sequences did not closely match pathogens. Furthermore, the sampled plants appeared healthy, raising questions about the potential for some of these viruses to be neutral or perhaps beneficial under some conditions. This study successfully characterized putative endophytic viromes from sympatric banana plants, showing differences among host tissues and genotypes with largely non-overlapping viromes, suggesting plant hosts drive or limit viral community structure. Future studies with long-read approaches should better-uncover longer regions to help distinguish active virions from integrated prophages or endogenous plant dsDNA viruses. Importantly, our data suggest that a Nycodenz-based microbiome enrichment method should make such long-read sequencing approaches more cost-effective for *in planta* virus community analysis.

## Data availability statement

The names of the repository/repositories and accession number(s) can be found in NCBI BioProject PRJNA837781, BioSamples SAMN28230877 to SAMN28230890.

## Author contributions

AB led the sample collection and sequencing. SA and RL developed and ran bioinformatic analyses pipelines. SA and AB drafted the manuscript. All authors contributed to the article and approved the submitted version.

## Funding

This research was funded by graduate funding support to SA through the Texas Tech Association of Biologists, Tech ASM, the TTU Graduate Student Research Award, Biology Graduate Summer Research Award, and the Helen DeVitt Jones Graduate Fellowship.

## Conflict of interest

The authors declare that the research was conducted in the absence of any commercial or financial relationships that could be construed as a potential conflict of interest.

## Publisher’s note

All claims expressed in this article are solely those of the authors and do not necessarily represent those of their affiliated organizations, or those of the publisher, the editors and the reviewers. Any product that may be evaluated in this article, or claim that may be made by its manufacturer, is not guaranteed or endorsed by the publisher.

## Supplementary material

SUPPLEMENTARY TABLE S1 List of detected putative phage and endogenous species from each sample, non-normalized and normalized read counts.

SUPPLEMENTARY TABLE S4 Predicted genes and protein motifs (pfams) using the DRAM-v software.

SUPPLEMENTARY TABLE S6 List of putative endogenous plant viruses detected from *Musa* spp. with taxonomic data and associated NCBI metadata.
