# Supplementary material for: Divergent endophytic viromes and phage genome repertoires among banana (Musa) species
Source: Front Microbiol. 2023 Jun 9;14:1127606. doi: 10.3389/fmicb.2023.1127606 (PMC10288200; doi:10.3389/fmicb.2023.1127606)
Supplement: Supplementary file 1 [file Data_Sheet_1.zip › Data Sheet 1.DOCX]

Supplementary Material

**Divergent endophytic viromes and phage genome repertoires among banana (*Musa*) species**

Shiva A. Aghdam, Rachel M. Lahowetz, Amanda M.V. Brown^*^

*** Correspondence:** Corresponding Author: amanda.mv.brown@ttu.edu

**
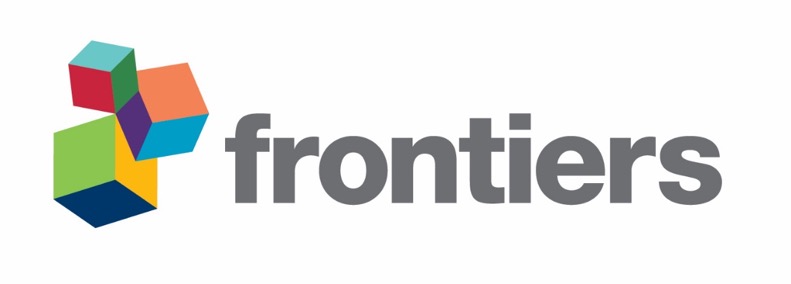
**

**Supplementary Table 1:** List of detected putative phage and endogenous species from each sample, non-normalized and normalized read counts.

(See separate uploaded excel file)

**Supplementary Table 2**: Trimmed and filtered sequence reads and metagenomic assembly statistics for *Musa* spp. extracted microbiome layers. (Samples: BBC & BBL = *Musa balbisiana* Thai Black corm & leaf, DCR & DCL = Dwarf Cavendish root, & leaf, FHC & FHL = FHIA-25 corm & leaf, MBR & MBL = *Musa balbisiana* root & leaf, MSR & MSL = *Musa sikkimensis* root & leaf, MTC & MTL = *Musa textilis* corm & leaf, WHC & WHL = Williams Hybrid corm & leaf).

| Sample ID | Number of raw reads after pairing (assembled and unassembled reads) | Largest contig length (bp) | Number of contigs in assembly | Assembly N50 | Reads mapped to *Musa* spp. (used to normalize endogenous virus coverage) | Reads not mapped to *Musa* spp. (used to normalize phage coverage) |
| --- | --- | --- | --- | --- | --- | --- |
| BBC | 70,191,820 | 527,409 | 373,469 | 1724 | 4,241,500 | 65,950,320 |
| BBL | 66,109,853 | 663,961 | 393,398 | 5334 | 51,351,379 | 14,758,474 |
| DCR | 38,564,855 | 758,840 | 455,793 | 881 | 7,560,618 | 31,004,237 |
| DCL | 39,144,858 | 88,717 | 782,984 | 919 | 33,939,965 | 5,204,893 |
| FHC | 64,298,991 | 726,320 | 238,899 | 1571 | 14,547,203 | 49,751,788 |
| FHL | 83,197,127 | 228,359 | 967,842 | 1223 | 62,683,988 | 20,513,139 |
| MBR | 60,216,239 | 381,654 | 446,178 | 797 | 12,209,729 | 48,006,510 |
| MBL | 52,880,189 | 465,474 | 255,235 | 9897 | 45,468,045 | 7,412,144 |
| MSR | 25,014,944 | 141,452 | 70,491 | 961 | 8,308,538 | 16,706,406 |
| MSL | 13,192,135 | 89,298 | 432,571 | 823 | 9,727,859 | 3,464,276 |
| MTC | 39,931,634 | 891,159 | 126,860 | 4102 | 912,113 | 39,019,521 |
| MTL | 23,309,361 | 153,218 | 444,694 | 1201 | 17,129,754 | 6,179,607 |
| WHC | 2,690,983 | 41,701 | 9,156 | 1878 | 497,889 | 2,193,094 |
| WHL | 1,275,593 | 24,295 | 4,901 | 670 | 470,636 | 804,957 |
| Total | 576,052,006 |  | 5,002,471 |  |  |  |

**Supplementary Table 3**: List of initial blastn-derived putative phage strains detected from *Musa* spp. using blastn to viral databases with the read coverage of each and the samples in which each strain was found. (Samples: BBC & BBL = *Musa balbisiana* Thai Black corm & leaf, DCR & DCL = Dwarf Cavendish root, & leaf, FHC & FHL = FHIA-25 corm & leaf, MBR & MBL = *Musa balbisiana* root & leaf, MSR & MSL = *Musa sikkimensis* root & leaf, MTC & MTL = *Musa textilis* corm & leaf, WHC & WHL = Williams Hybrid corm & leaf). Percent read coverage is relative to total read coverage for predicted phages.

| Closest phage species or strain match | % of read coverage | Samples |
| --- | --- | --- |
| Uncultured *Caudovirales* bacteriophage | 11.95 | BBC, BBL, MBR, MSL |
| *Rhizobium* bacteriophage RR1-A | 10.46 | BBC, MBR, MTC, DCR, FHC, FHL |
| *Rhizobium* bacteriophage RR1-B | 8.51 | MBR, MTC, DCR |
| *Klebsiella* bacteriophage ST13-OXA48phi12.4 | 8.35 | MSL, MTC |
| *Agrobacterium* bacteriophage Atu_ph08 | 6.16 | BBC |
| *Salmonella* bacteriophage SSU5 | 5.52 | BBL |
| *Pseudomonas* virus KNP | 4.07 | MSL |
| *Erwinia* bacteriophage EtG | 4.01 | BBC |
| *Enterobacter* bacteriophage phiT5282H | 3.35 | MTC, FHC, FHL |
| *Pseudomonas* bacteriophage AF | 3.16 | MTL |
| *Pectobacterium* bacteriophage ZF40 | 2.74 | MSR, MTC |
| *Burkholderia* virus DC1 | 2.54 | MTL |
| *Klebsiella* bacteriophage ST405-OXA48phi1.3 | 1.83 | MBR, MTC, FHC |
| *Escherichia* virus N15 | 1.67 | MTC |
| *Klebsiella* bacteriophage KOX7 | 1.58 | MTC |
| *Aeromonas* virus phiO18P | 1.45 | DCR |
| *Enterobacteria* bacteriophage HK106 | 1.30 | MTC |
| *Curvibacter* bacteriophage TJ1 | 1.26 | BBC, MSR |
| *Escherichia* bacteriophage PHB10 | 1.11 | BBC, MTC, DCR |
| *Erwinia* bacteriophage Midgardsormr38 | 1.08 | MBL |
| *Ralstonia* bacteriophage RsoM1USA | 0.95 | MSR |
| *Escherichia* bacteriophage ECP1 | 0.81 | MTC |
| *Arthrobacter* bacteriophage Seahorse | 0.73 | MSR |
| Prokaryotic dsDNA virus sp. | 0.7 | BBC |
| *Escherichia* bacteriophage mEpX2 | 0.68 | MTC |
| *Burkholderia* bacteriophage KS14 | 0.66 | BBC, DCR |
| *Enterobacter* bacteriophage Tyrion | 0.65 | MTC |
| *Gordonia* bacteriophage Bakery | 0.65 | DCR |
| *Stenotrophomonas* bacteriophage Smp131 | 0.64 | BBC |
| *Klebsiella* bacteriophage ST11-OXA245phi3.2 | 0.63 | MTC |
| *Caulobacter* bacteriophage Jess A | 0.59 | DCR |
| *Enterobacterial* bacteriophage mEp390 | 0.56 | MTC |
| *Rhizobium* bacteriophage RHEph10 | 0.55 | BBC |
| *Erwinia* bacteriophage phiEt88 | 0.53 | MTC |
| *Klebsiella* bacteriophage 020009 | 0.5 | MSR |
| *Escherichia* bacteriophage ESSI2 | 0.47 | MTC |
| *Aeromonas* bacteriophage phiARM81mr | 0.46 | DCR |
| *Klebsiella* bacteriophage ST405-OXA48phi1.2 | 0.45 | BBC, FHC |
| *Klebsiella* bacteriophage ST15-VIM1phi2.1 | 0.44 | MTC |
| *Microcystis* bacteriophage MACPNOA1 | 0.44 | MBR |
| *Ochrobactrum* bacteriophage POA1180 | 0.44 | BBC, MBR |
| *Escherichia* virus Lambda | 0.38 | MTC |
| *Klebsiella* bacteriophage ST846-OXA48phi9.2 | 0.38 | MTC |
| *Rhodobacter* bacteriophage RC1 | 0.36 | BBC |
| *Edwardsiella* bacteriophage GF-2 | 0.32 | DCR |
| *Mycobacterium* bacteriophage Kratio | 0.31 | BBC |
| *Salmonella* bacteriophage 7t3 | 0.27 | DCR |
| *Inoviridae* sp. | 0.27 | MBR |
| *Klebsiella* bacteriophage ST16-OXA48phi5.3 | 0.24 | FHC |
| *Yersinia* bacteriophage YeP5;*Yersinia* bacteriophage YeP6 | 0.23 | FHC |
| *Klebsiella* bacteriophage KPP5665-2 | 0.22 | BBC |
| *Rhizobium* bacteriophage vB_RleM_PPF1 | 0.21 | MBR |
| *Gordonia* bacteriophage PhorbesPhlower | 0.2 | BBC |
| *Pseudomonas* bacteriophage vB_Pae_BR319a | 0.2 | BBC |
| *Pseudomonas* bacteriophage vB_PaeP_E220 | 0.19 | FHC |
| *Pseudomonas* bacteriophage AAT-1 | 0.19 | BBC |
| *Ralstonia* bacteriophage GP4 | 0.18 | BBC |
| *Pseudomonas* virus phiCTX | 0.16 | BBC |
| *Bacillus* bacteriophage PBP180 | 0.16 | BBC |
| *Escherichia* bacteriophage SH2026Stx1 | 0.15 | BBC |
| *Erwinia* bacteriophage ENT90 | 0.14 | BBC |
| *Klebsiella* bacteriophage ST899-OXA48phi17.2 | 0.14 | FHC |
| *Pseudomonas* bacteriophage phi297 | 0.12 | BBC |

**Supplementary Table 4:** Predicted genes and protein motifs (pfams) using the DRAM-v software.

(See separate uploaded excel file)

**Supplementary Figure 1**. Phylogeny of predicted DNA methylase homologs for *Rhizobium*-like phage regions 56 and 68 from *Musa* microbiome samples BBC, MTC, FHL, FHC, based on 1,086 aligned amino acid positions, generated with maximum likelihood in RAxML with the GTR+Gamma model, showing bootstrap support from 1000 replicates on branches.

**Supplementary Figure 2**. Phylogeny of predicted DNA primase homologs for *Rhizobium*-like phage region 56 from *Musa* microbiome samples BBC and MTC, based on 608 aligned amino acid positions, generated with maximum likelihood in RAxML with the GTR+Gamma model, showing bootstrap support from 1000 replicates on branches.

**Supplementary Figure 3**. Phylogeny of predicted C-5 cytosine-specific DNA methylase homologs for *Rhizobium*-like phage regions 68 from *Musa* microbiome samples FHL and FHC, based on 819 aligned amino acid positions, generated with maximum likelihood in RAxML with the GTR+Gamma model, showing bootstrap support from 1000 replicates on branches.

**Supplementary Figure 4**. Phylogeny of predicted integrase arm-type DNA binding domain protein for *Agrobacterium* Atu-ph08-like phage region 88 from *Musa* microbiome samples BBC and MBR, based on 435 aligned amino acid positions, generated with maximum likelihood in RAxML with the GTR+Gamma model, showing bootstrap support from 1000 replicates on branches.

**Supplementary Figure 5**. Phylogeny of predicted UvrB/UvrC motif containing protein homologs for *Agrobacterium* Atu-ph08-like phage region 88 from *Musa* microbiome samples BBC and MBR, based on 321 aligned amino acid positions, generated with maximum likelihood in RAxML with the GTR+Gamma model, showing bootstrap support from 1000 replicates on branches.

**Supplementary Figure 6**. Phylogeny of putative internal core protein homologs for *Klebsiella*-like phage region 113 from *Musa* microbiome sample MTC, based on 894 aligned amino acid positions, generated with maximum likelihood in RAxML with the GTR+Gamma model, showing bootstrap support from 1000 replicates on branches.

**Supplementary Figure 7**. Phylogeny of predicted putative internal core protein homologs for *Klebsiella*-like phage region 113 from *Musa* microbiome sample MTC, based on 1,236 aligned amino acid positions, generated with maximum likelihood in RAxML with the GTR+Gamma model, showing bootstrap support from 1000 replicates on branches.

**Supplementary Figure 8**. Phylogeny of predicted hypothetical protein homologs for *Agrobacterium* Atu-ph08-like phage region 114 from *Musa* microbiome sample BBC, based on 767 aligned amino acid positions, generated with maximum likelihood in RAxML with the GTR+Gamma model, showing bootstrap support from 1000 replicates on branches.

**Supplementary Figure 9**. Phylogeny of predicted hypothetical protein homologs for *Agrobacterium* Atu-ph08-like phage region 114 from *Musa* microbiome sample BBC, based on 512 aligned amino acid positions, generated with maximum likelihood in RAxML with the GTR+Gamma model, showing bootstrap support from 1000 replicates on branches.

**Supplementary Figure 10**. Phylogeny of predicted peptidoglycan-binding domain protein homologs for *Shinella*-like phage regions 116 from *Musa* microbiome sample MTC, based on 363 aligned amino acid positions, generated with maximum likelihood in RAxML with the GTR+Gamma model, showing bootstrap support from 1000 replicates on branches.

**Supplementary Figure 11**. Phylogeny of predicted transposase C-terminal domain-containing protein homologs for *Shinella*-like phage regions 116 from *Musa* microbiome sample MTC, based on 751 aligned amino acid positions, generated with maximum likelihood in RAxML with the GTR+Gamma model, showing bootstrap support from 1000 replicates on branches.

**Supplementary Figure 12**. Phylogeny of predicted phage tail tape measure protein homologs for *Rhizobium*-like phage region ‘DCR phage’ from *Musa* microbiome sample DCR, based on 748 aligned amino acid positions, generated with maximum likelihood in RAxML with the GTR+Gamma model, showing bootstrap support from 1000 replicates on branches.

**Supplementary Figure 13**. Phylogeny of predicted head maturation protease homologs for *Rhizobium*-like phage region ‘DCR phage’ from *Musa* microbiome sample DCR, based on 713 aligned amino acid positions, generated with maximum likelihood in RAxML with the GTR+Gamma model, showing bootstrap support from 1000 replicates on branches.

**Supplementary Figure 14**. Phylogeny of predicted phage tail tape measure protein homologs for *Pseudomonas*-like phage region ‘FHC phage’ from *Musa* microbiome sample FHC, based on 982 aligned amino acid positions, generated with maximum likelihood in RAxML with the GTR+Gamma model, showing bootstrap support from 1000 replicates on branches.

**Supplementary Figure 15**. Phylogeny of predicted hypothetical protein homologs for *Pseudomonas*-like phage region ‘FHC phage’ from *Musa* microbiome sample FHC, based on 1,348 aligned amino acid positions, generated with maximum likelihood in RAxML with the GTR+Gamma model, showing bootstrap support from 1000 replicates on branches.

**Supplementary Table 5**: Names of predicted proteins shown in Figure 6. Note: additional predicted proteins with unknown function (hypothetical proteins) are not numbered).

| **Predicted Phage** | **Number** | **Predicted product** |
| --- | --- | --- |
| *Musa* microbiome phage 56 | 1 | DNA polymerase III subunit beta |
|  | 2 | single-stranded DNA-binding protein |
|  | 3 | outer capsid protein lambda-2 |
|  | 4 | DNA methylase |
|  | 5 | C-5 cytosine-specific DNA methylase |
|  | 6 | phosphoadenosine phosphosulfate reductase |
|  | 7 | DNA primase |
| *Musa* microbiome phage 68 | 1 | ISL3 family transposase |
|  | 2 | Shiga-like toxin 2 subunit A |
|  | 3 | tail length tape-measure protein |
|  | 4 | DNA polymerase III subunit beta |
|  | 5 | single-stranded DNA-binding protein |
|  | 6 | repressor protein C |
|  | 7 | DNA methylase |
|  | 8 | C-5 cytosine-specific DNA methylase |
|  | 9 | DNA primase |
|  | 10 | terminase, large subunit (tail assembly) |
|  | 11 | portal protein B |
|  | 12 | peptidase S14 |
|  | 13 | tail sheath protein |
|  | 14 | phage tail tape measure |
|  | 15 | holin |
|  | 16 | hypothetical Rha protein |
|  | 17 | baseplate hub protein gp44 |
|  | 18 | baseplate protein gp47 |
| *Rhizobium* phage RR1-B (JF974315.1) | 1 | terminase large subunit |
|  | 2 | portal protein |
|  | 3 | peptidase S14 |
|  | 4 | tail sheath protein |
|  | 5 | tail protein |
|  | 6 | baseplate J family protein |
|  | 7 | peptidoglycan-binding domain 1 protein |
|  | 8 | recombinase |
|  | 9 | DNA polymerase III subunit beta |
|  | 10 | single-stranded DNA-binding protein |
|  | 11 | DNA methylase |
|  | 12 | C-5 cytosine-specific DNA methylase |
|  | 13 | phosphoadenosine phosphosulfate reductase |
|  | 14 | DNA primase |
|  | 15 | antirepressor |
| *Musa* microbiome phage 114 | 1 | C-5 cytosine-specific DNA methylase |
|  | 2 | putative PRK12775-containing protein |
|  | 3 | tail fibers protein |
|  | 4 | DNA adenine methyltransferase |
| *Musa* microbiome phage 88 | 1 | integrase arm-type DNA binding domain protein |
|  | 2 | CzcR-like response regulator |
|  | 3 | endolysin |
| *Agrobacterium* phage Atu ph08 (MF403009.1) | 1 | C-5 cytosine-specific DNA methylase |
|  | 2 | capsid decoration protein |
|  | 3 | putative PRK12775-containing protein |
|  | 4 | putative transcriptional regulator |
|  | 5 | putative DNA methylase |
|  | 6 | virion associated protein |
|  | 7 | putative tail fiber assembly-like protein |
|  | 8 | virion associated protein |
|  | 9 | major capsid protein |
|  | 10 | genome polyprotein |
|  | 11 | ATP-dependent DNA helicase dda |
| *Musa* microbiome phage 113 | 1 | methyltransferase type 11 |
|  | 2 | gag polyprotein |
|  | 3 | replicase polyprotein 1a |
|  | 4 | endolysin |
|  | 5 | endolysin |
|  | 6 | nucleotide kinase gp1.7 |
|  | 7 | RNA-directed RNA polymerase L |
|  | 8 | T7 RNA polymerase |
|  | 9 | portal protein |
|  | 10 | putative phosphoesterase |
|  | 11 | envelope glycoprotein |
|  | 12 | putative tail fiber protein |
|  | 13 | terminase, large subunit gp19 |
|  | 14 | U-spanin |
|  | 15 | tail fiber protein |
| *Klebsiella* phage KMI6 (MN101220.1) | 1 | DNA packaging protein (terminase, large subunit gp19) |
|  | 2 | tail assembly protein |
|  | 3 | U-spanin |
|  | 4 | putative single-stranded DNA binding protein |
|  | 5 | pore-forming tail tip protein |
|  | 6 | methyltransferase type 11 |
|  | 7 | HNH endonuclease |
|  | 8 | DNA primase/helicase |
|  | 9 | putative DNA polymerase |
|  | 10 | DNA polymerase I |
|  | 11 | hemagglutinin-neuroaminidase |
|  | 12 | putative DNA endonuclease VII |
|  | 13 | nucleotide kinase gp1.7 |
|  | 14 | T7 RNA polymerase |
|  | 15 | putative HNH endonuclease |
|  | 16 | portal protein |
|  | 17 | putative scaffolding protein |
|  | 18 | holin |
|  | 19 | holin |
|  | 20 | tail fibers protein |
|  | 21 | tail tubular protein B |
|  | 22 | putative internal virion protein |
|  | 23 | primase |
| *Musa* microbiome phage 116 | 1 | predicted N-acetylmuramoyl-L-alanine amidase |
|  | 2 | GemA protein |
|  | 3 | conserved transposable phage protein |
|  | 4 | host-nuclease inhibitor protein |
|  | 5 | putative DNA ends protecting protein gam |
|  | 6 | transposase |
|  | 7 | chromosome partitioning protein parB |
|  | 8 | repressor protein CI |
| *Shinella* sp. HZN7 (CP015736.1) | 1 | lysozyme |
|  | 2 | transposase |
|  | 3 | chromosome partitioning protein parB |
|  | 4 | repressor |
| *Musa* microbiome phage FHC | 1 | ParA-like protein |
|  | 2 | tail protein |
|  | 3 | putative tail protein |
|  | 4 | putative tail tape measure |
|  | 5 | putative tail tube protein |
|  | 6 | major tail sheath protein |
|  | 7 | tail fiber protein |
|  | 8 | putative tail fiber protein |
|  | 9 | putative tail protein |
|  | 10 | putative tail protein |
|  | 11 | baseplate assembly protein |
|  | 12 | putative baseplate assembly protein |
|  | 13 | putative baseplate assembly protein V |
|  | 14 | major capsid protein |
|  | 15 | portal protein |
|  | 16 | primase |
|  | 17 | putative terminase large subunit |
|  | 18 | putative terminase small subunit |
|  | 19 | methyltransferase type 11 |
|  | 20 | gp32 |
|  | 21 | tail fiber protein |
|  | 22 | transcription antiterminator |
|  | 23 | prophage antirepressor |
|  | 24 | prophage repressor |
|  | 25 | replication protein RepA |
|  | 26 | protelomerase |
| *Pseudomonas monteilii* plasmid pSTW0522-71-1 (AP022474.1) | 1 | ParA-like protein |
|  | 2 | tail protein |
|  | 3 | phage tail X |
|  | 4 | putative tail protein |
|  | 5 | putative tail tape measure |
|  | 6 | putative tail tube protein |
|  | 7 | major tail sheath protein |
|  | 8 | primase |
|  | 9 | tail protein |
|  | 10 | putative tail protein |
|  | 11 | baseplate assembly protein |
|  | 12 | putative baseplate assembly protein |
|  | 13 | putative baseplate assembly protein V |
|  | 14 | major capsid protein |
|  | 15 | portal protein |
|  | 16 | putative terminase large subunit |
|  | 17 | putative terminase small subunit |
|  | 18 | tail fiber protein |
|  | 19 | envelope glycoprotein |
|  | 20 | primase |
|  | 21 | transcription antiterminator |
|  | 22 | prophage antirepressor |
|  | 23 | putative repressor protein |
|  | 24 | replication protein RepA |
|  | 25 | protelomerase |

**Supplementary Table 6**: List of putative endogenous plant viruses detected from Musa spp. with taxonomic data and associated NCBI metadata (see attached excel file). (Samples: BBC & BBL = *Musa balbisiana* Thai Black corm & leaf, DCR & DCL = Dwarf Cavendish root, & leaf, FHC & FHL = FHIA-25 corm & leaf, MBR & MBL = *Musa balbisiana* root & leaf, MSR & MSL = *Musa sikkimensis* root & leaf, MTC & MTL = *Musa textilis* corm & leaf, WHC & WHL = Williams Hybrid corm & leaf).

**Supplementary Table 7**: List of putative endogenous plant viruses detected from *Musa* spp. using blastn to viral databases with the read coverage of each and the samples in which each strain was found. (Samples: BBC & BBL = *Musa balbisiana* Thai Black corm & leaf, DCR & DCL = Dwarf Cavendish root, & leaf, FHC & FHL = FHIA-25 corm & leaf, MBR & MBL = *Musa balbisiana* root & leaf, MSR & MSL = *Musa sikkimensis* root & leaf, MTC & MTL = *Musa textilis* corm & leaf, WHC & WHL = Williams Hybrid corm & leaf). Percent read coverage is relative to total read coverage for predicted endogenous viruses.

| Putative endogenous virus species | % of read coverage | Samples |
| --- | --- | --- |
| Banana streak virus | 26.20 | BBC, BBL, MBR, MBL, MTC, MTL, MSR, MSL, DCR, DCL, FHC, FHL |
| *Musa* *balbisiana* endogenous badnavirus | 15.42 | BBC, BBL, MBR, MBL, MTC, MTL, MSR, MSL, DCR |
| *Musa* ABB Group endogenous badnavirus | 12.08 | BBC, BBL, MBR, MBL, MTL, MSL, DCR, FHL |
| Banana streak MY virus | 9.15 | BBC, BBL, MBR, MBL, MTL, FHC, FHL |
| *Musa* *acuminata* subsp. *malaccensis* endogenous badnavirus | 4.25 | MSR, MSL, DCR, DCL, FHC, FHL |
| *Musa* *acuminata* subsp. *burmannicoides* endogenous badnavirus | 3.73 | MSR, MSL, DCR, DCL, FHL |
| *Musa* AAB Group endogenous badnavirus | 3.01 | BBC, MBR, MBL, MTL, MSR, DCR, DCL, FHC, FHL |
| Grapevine Roditis leaf discoloration-associated virus | 2.52 | BBC, MBR, MBL, MTL, MSL, DCL, FHC, FHL |
| Fig badnavirus 1 | 2.35 | MSR, MSL |
| Banana streak OL virus | 2.04 | BBC, BBL, MBL, MTL |
| Taro bacilliform CH virus | 1.93 | BBL, MBR, MBL, MTL, MSR, MSL, DCL, FHC, FHL |
| Yacon necrotic mottle virus | 1.87 | MSR, MSL |
| *Musa* sp. IBo01 endogenous badnavirus | 1.55 | DCR, DCL, FHC, FHL |
| *Musa* *itinerans* endogenous badnavirus | 1.51 | MSR, MSL |
| Banana streak GF virus | 1.18 | BBL, MBR, MBL, MTL, FHC |
| Banana streak Uganda E virus | 0.95 | DCR, DCL |
| *Musa* *banksii* endogenous badnavirus | 0.85 | MSL, DCL, FHC |
| Hibiscus bacilliform virus GD1 | 0.81 | MSL |
| *Musa* *acuminata* endogenous badnavirus | 0.7 | MSL, DCR, DCL, FHC, |
| Banana streak IM virus | 0.63 | BBL, MBR, MBL, MTL, MSR, MSL, FHL |
| Banana streak virus acuminata Yunnan | 0.56 | BBL, MBR, MBL, MTL, MSR, MSL, FHL |
| Cacao swollen shoot Ghana Q virus | 0.53 | MSL, FHL |
| *Musa* *yunnanensis* endogenous badnavirus | 0.41 | MSL |
| Piper yellow mottle virus | 0.41 | BBL, MBR, MBL, MTL |
| Banana streak UL virus | 0.40 | MSL |
| Green huajiao vein-clearing badnavirus | 0.38 | MSR, |
| Cacao swollen shoot Ghana R virus | 0.37 | MBR, MBL, FHL |
| Grapevine vein clearing virus | 0.36 | DCL, FHL |
| *Musa* *schizocarpa* endogenous badnavirus | 0.34 | DCL, FHC |
| Banana streak Uganda H virus | 0.33 | DCR |
| *Musa* *acuminata* var. zebrina endogenous badnavirus | 0.3 | DCR, DCL |
| Sugarcane bacilliform virus | 0.29 | BBC, MBR, MBL, MTL |
| Sugarcane bacilliform Guadeloupe A virus | 0.28 | BBL, MBR, MBL, MTL, FHL |
| Banana streak VN virus | 0.27 | BBL, MBL, FHL |
| Cacao swollen shoot virus | 0.15 | MSR |
| Dioscorea bacilliform AL virus 2 | 0.13 | MSR |
| Bougainvillea chlorotic vein banding virus | 0.13 | MBR, MTL |
| Banana streak Uganda G virus | 0.12 | DCR, DCL |
| Dioscorea bacilliform virus | 0.12 | MTL, MSR |
| *Musa* *balbisiana*; *Musa acuminata* subsp. *siamea* endogenous badnavirus | 0.11 | BBC |
| Banana streak Uganda B virus | 0.1 | DCR |
| Banana streak Uganda virus C | 0.1 | DCR, DCL |
| Banana streak UI virus | 0.09 | MBR |
| Dioscorea bacilliform RT virus 1 | 0.09 | MBR |
| Banana streak CA virus | 0.09 | BBL, MTL |
| Dioscorea bacilliform TR virus | 0.08 | MSL |
| Pineapple bacilliform CO virus | 0.08 | MTL |
| Dioscorea bacilliform RT virus 2 | 0.07 | BBL |
| Banana streak brazilian B virus | 0.06 | MBL |
| *Musa* *acuminata* subsp. *siamea* endogenous badnavirus | 0.06 | DCL |
| Grapevine badnavirus 1 | 0.05 | FHC |
| Cacao red vein-banding virus | 0.04 | DCL, FHL |
| Banana streak UA virus | 0.03 | MTL, |
| Canna yellow mottle virus | 0.03 | MBL |
| Birch leaf roll-associated virus | 0.01 | FHL |
| Enset leaf streak virus | 0.008 | FHL |
